# Engineering CAR-T cells for radiohapten capture in imaging and radioimmunotherapy applications

**DOI:** 10.7150/thno.87489

**Published:** 2023-10-08

**Authors:** Keifer Kurtz, Laura Eibler, Megan M. Dacek, Lukas M. Carter, Darren R. Veach, Samantha Lovibond, Emma Reynaud, Sarah Qureshy, Michael R. McDevitt, Christopher Bourne, Sebastien Monette, Blesida Punzalan, Shireen Khayat, Svena Verma, Adam L. Kesner, Nai-Kong V. Cheung, Heiko Schöder, Leah Gajecki, Sarah M. Cheal, Steven M. Larson, David A. Scheinberg, Simone Krebs

**Affiliations:** 1 Molecular Pharmacology Program, Sloan Kettering Institute, Memorial Sloan Kettering Cancer Center, New York, NY 10065, USA.; 2 Department of Pharmacology, Weill Cornell Medical College, New York, NY 10065, USA.; 3 Molecular Imaging and Therapy Service, Department of Radiology, Memorial Sloan Kettering Cancer Center, New York, NY 10065, USA.; 4 Department of Medical Physics, Memorial Sloan Kettering Cancer Center, New York, NY 10065, USA.; 5 Radiochemistry and Imaging Sciences Service, Department of Radiology, Memorial Sloan Kettering Cancer Center, New York, NY 10065, USA.; 6 Department of Radiology, Weill Cornell Medical College, New York, NY 10065, USA.; 7 Immunology and Microbial Pathogenesis Program, Weill Cornell Medical College, New York, NY 10065, USA.; 8 Laboratory of Comparative Pathology, Memorial Sloan Kettering Cancer Center, Weill Cornell Medicine, and The Rockefeller University, New York, NY 10065, USA.; 9 Department of Pediatrics, Memorial Sloan Kettering Cancer Center, New York, NY 10065, USA.; ##Contributed equally as senior authors.

**Keywords:** CAR-T cells, reporter gene, T cell tracking, theranostic, alpha-particles

## Abstract

**Rationale:** The *in vivo* dynamics of CAR-T cells remain incompletely understood. Novel methods are urgently needed to longitudinally monitor transferred cells non-invasively for biodistribution, functionality, proliferation, and persistence *in vivo* and for improving their cytotoxic potency in case of treatment failure.

**Methods:** Here we engineered CD19 CAR-T cells (“Thor”-cells) to express a membrane-bound scFv, huC825, that binds DOTA-haptens with picomolar affinity suitable for labeling with imaging or therapeutic radionuclides. We assess its versatile utility for serial tracking studies with PET and delivery of α-radionuclides to enhance anti-tumor killing efficacy in sub-optimal adoptive cell transfer *in vivo* using Thor-cells in lymphoma models.

**Results:** We show that this reporter gene/probe platform enables repeated, sensitive, and specific assessment of the infused Thor-cells in the whole-body using PET/CT imaging with exceptionally high contrast. The uptake on PET correlates with the Thor-cells on a cellular and functional level. Furthermore, we report the ability of Thor-cells to accumulate cytotoxic alpha-emitting radionuclides preferentially at tumor sites, thus increasing therapeutic potency.

**Conclusion:** Thor-cells are a new theranostic agent that may provide crucial information for better and safer clinical protocols of adoptive T cell therapies, as well as accelerated development strategies.

## Introduction

CD19-targeted chimeric antigen receptor (CAR) T cell therapy has shown remarkable treatment effects in B-cell malignancies, but many patients suffer from limited response and CD19-negative tumor relapse 1-3. Furthermore, success has been limited in patients with solid tumors 4, 5.

Several mechanisms contribute to the limitations of CAR-T therapy, including a lack of efficacy resulting from unsuccessful trafficking to the tumor site, reduced CAR-T persistence and immune effector function, relapse due to the loss of target antigen expression on tumor cells, and toxic side effects resulting from uncontrolled proliferation or “on-target/off-tumor” effects 6, 7. One hurdle for the advancement of CAR-T therapy is the almost total lack of understanding of the *in vivo* pharmacokinetics of CAR-T cells 8. Blood analyses and tumor biopsies do not provide the needed spatiotemporal information. The development of tools for non-invasive, real-time monitoring in the whole body and for improving the CAR-T cells' cytotoxic potency in case of treatment failure is required to advance CAR-T therapies.

Positron emission tomography (PET) and single-photon emission computed tomography (SPECT), co-registered with CT or MRI, enable non-invasive, repetitive, and quantitative imaging of radiotracers with picomolar sensitivity suitable for whole-body visualization of cell trafficking 9. While multiple reporter-based PET approaches have been studied in preclinical models, herpes simplex virus type 1-thymidine kinase (HSV1-tk) is the only reporter gene reported in patients 10. Furthermore, HSV1-tk is a viral protein, and immune reactions have been observed 10. Risk of immunogenic response of transduced cells can be reduced by exploiting naturally expressed genes as reporters, such as human prostate-specific membrane antigen (PSMA) 11 and somatostatin receptor 2 (SSTR) 12, 13, but their physiologic expression pattern may limit the organ systems that can be studied.

We reported the use of a T cell membrane-bound 1,4,7,10-tetraazacyclo-dodecane-1,4,7,10-tetraacetic acid (DOTA) antibody reporter 1 (DAbR1) that is capable of binding DOTA-radiohaptens and enables visualization of migrated tumor-targeted T cells at the tumor site 14. DOTA is a chelator that is used clinically as part of MRI contrast agents and radiopharmaceuticals. However, DAbR1 is of murine origin, which limits its application in humans. Furthermore, the binding affinity to the radiohapten is low, requiring the introduction of a covalent bond, and the excretion of the probe is primarily hepatobiliary, limiting assessment of the abdominal cavity on early images. Therefore, we sought to equip CAR-T cells with the picomolar affinity anti-benzyl-DOTA scFv, C825, which has been humanized (huC825), in conjunction with preferentially renally clearing radiohaptens. The scFv and the haptens have been used for pretargeting 15. Here, we adapted this technology into a novel CAR-T cell platform. We investigated its utility for tracking *in vivo*, and systematically addressed the sensitivity and specificity of the radiotracer uptake by PET of the T cell infiltrates in the tumors and tissues. Finally, we show that Thor-cells can be repurposed to bind a cytotoxic radiohapten payload and destroy tumor cells in a manner orthogonal to the T cell function, which may increase the therapeutic potential of CAR-T therapy. The Thor-cells fulfill the requirements for a clinically relevant reporter system and represent a promising new cellular theranostic agent.

## Methods

### Retrovirus generation and human T cell transduction

H29 and Galv9 producer cells were generously provided by the Renier Brentjens lab. H29 packaging cells were transfected using CaPO_4_ (Promega) and the supernatant was used to generate stable Galv9 packaging cell lines. Human blood was collected from healthy donors in compliance with Memorial Sloan Kettering Cancer Center (MSK) IRB 00009377. Human PBMCs were isolated using Ficol gradient density separation; PBMCs were activated with anti-CD3 (50 ng/mL, clone, Miltenyi Biotec, 130-093-387) and IL-2 (100 IU/mL, MSKCC pharmacy) to generate T cells. IL-2 was replenished every other day to culture the T cells. On day 2 post-PBMC isolation, the T cells were transduced with retrovirus from the corresponding Galv9 supernatants harboring virus. T cell transduction was performed using RetroNectin (Takara)-coated plates and centrifugation (2000x g, 1 h). On day 3, transduction was repeated.

### Flow cytometry

Flow cytometry was used to determine the T cell transduction efficiency using Alexa-fluor647 conjugated anti-idiotype antibody that detects the CD19 CAR (clone 19E3, generated at Memorial Sloan Kettering Antibody Core Facility); huC825 transduction was measured using a biotinylated non-radioactive hapten probe [^nat^Lu]LuDOTA- PEG_6_-Biotin (Biotin-Pr, MSKCC Organic Synthesis Core Facility) and a streptavidin conjugated to Alexa-fluor488 or PE (BioLegend, 405235 and 405204). huC825 expression from CAR^+^, huCD3^+^ (ebioscience, 47-0038-42), huCD45^+^ (BioLegend, 304042) cells in the Raji co-culture system was detected using Biotin-Pr and a PE-linked streptavidin (BioLegend, 405204). Normalized MFI for each marker, huC825, CD25 (BioLegend, 302612), TIM3 (BioLegend, 345028), and LAG3 (BioLegend, 369345) in CAR^+^, huCD3^+^, huCD45^+^ huC825-19BBz cells from *ex vivo* samples were calculated as fold change over the corresponding FMO.

### *In vitro* cytotoxicity assay

Raji fluc/eGFP cells (5x10^5^) were co-cultured with various amounts of transduced CAR-T cells and control T cells to compare the anti-tumor effects of these cells. Total luminescence of Raji fluc/eGFP cells was measured using a D-luciferin substrate (GoldBio, 5 µL of a 20 mg/mL solution) and detected using a multimode plate reader (EnVision 2104, PerkinElmer). Percent specific lysis was calculated using Raji fluc/eGFP cells without T cells as a baseline measurement. Supernatants from these co-cultures were collected for cytokine secretion analysis using Luminex plate reader.

### Determination of *in vitro* uptake of the [^111^In]In-Pr, assessment of specificity, binding kinetics, and quantitation of binding sites

5x10^5^ transduced T cells were suspended in RPMI + 10% fetal calf serum, aliquoted into polystyrene culture tubes and treated with 0.1 nM or 1:5 serial dilutions of an 8 nM stock solution of [^111^In]In-Pr in PBS in a final volume of 500 μL. Tubes were incubated with gentle shaking at 37 °C for 1 h. Untransduced T cells served as a negative control. All experiments were conducted in triplicate. Nonspecific binding was measured at 3 or more ligand concentrations without cells. The binding curves were plotted, and equilibrium binding parameters (K_D_ and B_max_) computed by least-squares non-linear regression using a one-site total and nonspecific binding model implemented in GraphPad Prism 7 software (Graph Pad software, Inc., La Jolla, CA). No weighting was applied to the ordinate data, and model parameters corresponding to non-specific and background binding were shared between the data sets.

### *In vivo* experiments

All animal experiments were performed under a protocol approved by Memorial Sloan Kettering Cancer Center's Institutional Animal Care and Use Committee. To determine the ability to track huC825-19BBz cells *in vivo*, 3x10^6^ Raji cells were implanted subcutaneously over the right shoulder of female NSG mice (Jackson Laboratory). Seven days later, the mice were injected i.v. with either huC825 T cells, 19BBz T cells, or huC825-19BBz T cells (3x10^6^ transduced cells). On days 7, 14, 21, and 28 post-T cell injection, [^86^Y]Y-aminobenzyl-1,4,7,10-Tetraazacyclododecane-1,4,7,10-tetraacetic acid or [^86^Y]Y-aminobenzyl-DOTA, further referred to as [^86^Y]Y-ABD (3.7 MBq) was administered i.v. and the radiotracer uptake was measured by PET/CT (positron emission tomography / computerized tomography).

The *in vivo* efficacy of huC825-19BBz T cells combined with [^225^Ac]Ac-Pr was measured using the same tumor and T cell injection parameters as the *in vivo* tracking experiment. On day 7 post-T cell injection, 74 kBq of [^225^Ac]Ac-Pr was injected i.v. in both huC825-19BBz and 19BBz CAR-T cells or mice receiving no T cells. Tumor burden was measured in two ways: longitudinal BLI measurements from the day of [^225^Ac]Ac-Pr injection and with caliper measurement of tumor volume (TV) in mm^3^ using the ellipsoidal formula:

V=4/3π(length/2×width/2×height/2)

Mice were euthanized when the TV was greater than 2,000 mm^3^ in SC models or when they met euthanasia criteria (weight loss, signs of distress, ulcerated tumors, distended abdomens) in accordance with IACUC, OLAW, and AALAC guidelines.

### Small-animal PET/CT imaging

Small-animal PET/CT scans were performed using the Inveon PET/CT system (Siemens Manufacturing, Freeburg, IL). Mice were anesthetized using 1.5% - 2% isoflurane (Baxter Healthcare, Deerfield, IL) and intravenously injected with [^86^Y]Y-ABD (3.7 MBq). At day 14 each mouse underwent a 30 min scan at 1, 3, 16, and 36 h post-tracer injection. Furthermore, serial imaging was performed over the course of 4 weeks, with weekly µPET/CT scans on days 7, 14, 21, and 28 post-T cell injection at 16 h post-tracer injection. Data were corrected for decay and detector dead-time and images were reconstructed by 3D OSEM maximum a posteriori (2 OSEM iterations; 18 MAP iterations) into a 128 × 128 matrix (0.78 × 0.78 × 0.80 mm voxel dimensions). Image counts per voxel per second were converted to activity concentrations (Bq/mL) using a system-specific calibration factor and subsequently normalized by injected activity to units of percentage injected activity per gram (%ID/g) decay-corrected to time of injection. CT scans were reconstructed using a modified Feld Kamp cone beam reconstruction algorithm to generate 512 × 512 × 768 voxel image volumes (0.197 × 0.197 × 0.197 mm voxel dimensions).

### Dosimetry

Murine dosimetry estimates were obtained from [^86^Y]Y-ABD PET/CT images in order to 1) determine feasibility of ABD/Pr as a platform for molecular radiotherapy/theranostics and 2) to guide selection of administered activities when ABD/Pr is bound to different therapeutic radionuclides. An in-house program that utilizes the Particle and Heavy Ion Transport Code System (PHITS v. 3.20) was used to perform the murine dose calculations. Human dosimetry estimates were extrapolated from the murine [^86^Y]Y-ABD PET/CT images and computed with MIRDcalc software. Specific methods are detailed in the supplementary material.

### Statistical analysis

All experimental data are presented as mean ± standard deviation. All *in vitro* experiments were performed in triplicate linear regression analysis and Pearson correlation was performed for comparisons, and GraphPad Prism 7 software was used for statistical analysis. Differences between means were tested by appropriate tests, including t-tests.

## Results

### huC825-expressing T cells retain their effector functions and can bind DOTA-haptens *in vitro* and *in vivo*

**Figure 1:**
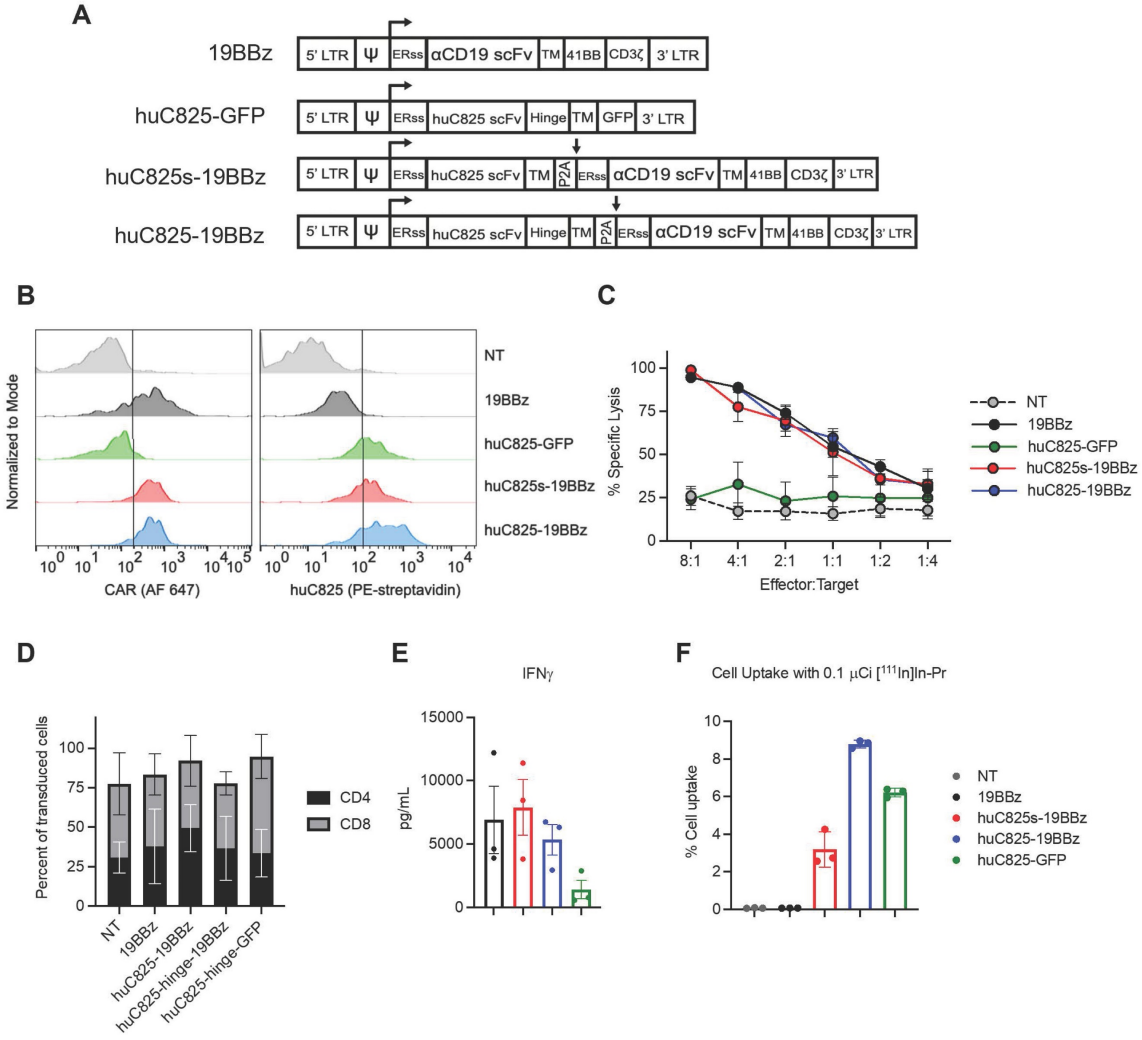
** huC825-expressing CAR-T cells are not altered in their effector functions and specifically bind DOTA-haptens *in vitro*.** (A) Schematic representation of retroviral vectors. (B) Transduction efficiency of CAR-T cells from (A) by flow cytometry is depicted for one representative donor using the 19E3 anti-CAR antibody (left) and Biotin-Pr for huC825 (right). (C) Killing of Raji tumor cells at different effector to target ratios at 24 h post co-culture as measured by total bioluminescence of the Raji tumor cells is shown. (D) The relative number of CD4^+^ and CD8^+^ cells from successfully transduced and non-transduced (NT) cells were measured by flow cytometry and compared. Mean ± S.D. is depicted for all. (E) Cytokine secretion levels as measured by Luminex are depicted using the co-culture supernatant from (C). (F) *In vitro* binding of [^111^In]In-Pr at 1 h from a representative data set, demonstrating the specific binding of the radiolabeled DOTA-hapten to huC825-expressing T cells, whereas no significant uptake was observed in NT and 19BBz T cells (reported as mean ± se for all). Data derived from at least three different donors for (C-F).

We generated a panel of CD19 CAR (19BBz) T cells expressing different linkers of huC825: one with a human IgG_4_-CH2CH3 hinge/spacer between the human CD4-transmembrane domain (TM) and the huC825 scFv (huC825-CAR), and one without a hinge between TM and the scFv (huC825s-CAR). In addition, we included T cells modified to express huC825 and GFP as well as standard 19BBz T cells (Figure 1A). Transduction efficiency was evaluated by flow cytometry using [^nat^Lu]LuDOTA- PEG_6_-Biotin (Biotin-Pr) capable of binding huC825 in combination with streptavidin-fluorophore 15 and the anti-CD19 19E3 antibody (generated by MSK's Antibody and Bioresource Core Facility) (Figure 1B). The intrinsic T cell effector functions of huC825-expressing CAR Ts were preserved; 19BBz Ts, huC825s-19BBz, and huC825-19BBz T cells showed similar cytolytic capacity against CD19^+^ Raji tumor cells *in vitro* (Figure 1C), demonstrating that T cell killing depends on the cell surface expression of the CD19 CAR and is not altered by the expression of huC825. No difference in CD4/CD8 subpopulations was observed (Figure 1D). Additionally, huC825-expressing CAR-T cells secreted comparable amounts of IFN-γ when compared to 19BBz T cells (Figure 1E).

In *in vitro* radiohapten binding assays, huC825-expressing T cells exhibited high accumulation of [^111^In]In-Pr, whereas uptake of the non-transduced and 19BBz T cells remained low (Figure 1F). Thus, transduced T cells expressed membrane-bound huC825, enabling specific binding of DOTA-hapten *in vitro*. To further characterize the kinetics of radiohapten capture *in vitro* and cell surface expression of membrane-bound huC825, we performed standard saturation binding assays. We determined a mean K_d_ (equilibrium dissociation constant) of 720 ± 400 pM, mean B_max_ (sites/cell) of 25000 ± 17000, mean R^2^ of 0.98 ± 0.01 for huC825s-CAR, mean K_d_ of 720 ± 340 pM, mean B_max_ of 76000 ± 48000, and mean R^2^ of 0.99 ± 0.01 for huC825-CAR, and a mean K_d_ of 720 ± 340 pM, mean B_max_ of 76000 ± 48000, and mean R^2^ of 0.99 ± 0.01 for huC825-GFP T cells (n = 3 donors) (Figure S1, Table S1). Thus, the incorporation of an IgG_4_-CH2CH3 hinge/spacer into the reporter design enabled binding of DOTA-haptens at a significantly higher level, while maintaining binding affinity. Therefore, we selected the huC825-19BBz construct for further characterization and henceforth refer to these cells as 'Thor-cells'.

*In vivo*, we observed no significant differences between Thor and CAR-T cells' antitumor activity and observed uptake of [^86^Y]Y-aminobenzyl-DOTA ([^86^Y]Y-ABD) at the tumor site using an intraperitoneal lymphoma mouse model (Figure S2).

Using titrated Thor-cell numbers in a T cell deposit assay, we determined a T cell detection limit of 30,000 cells per volume of 40 µL (Figure S3).

### The huC825-reporter gene/probe platform serves as a pharmacokinetic tool *in vivo*

**Figure 2:**
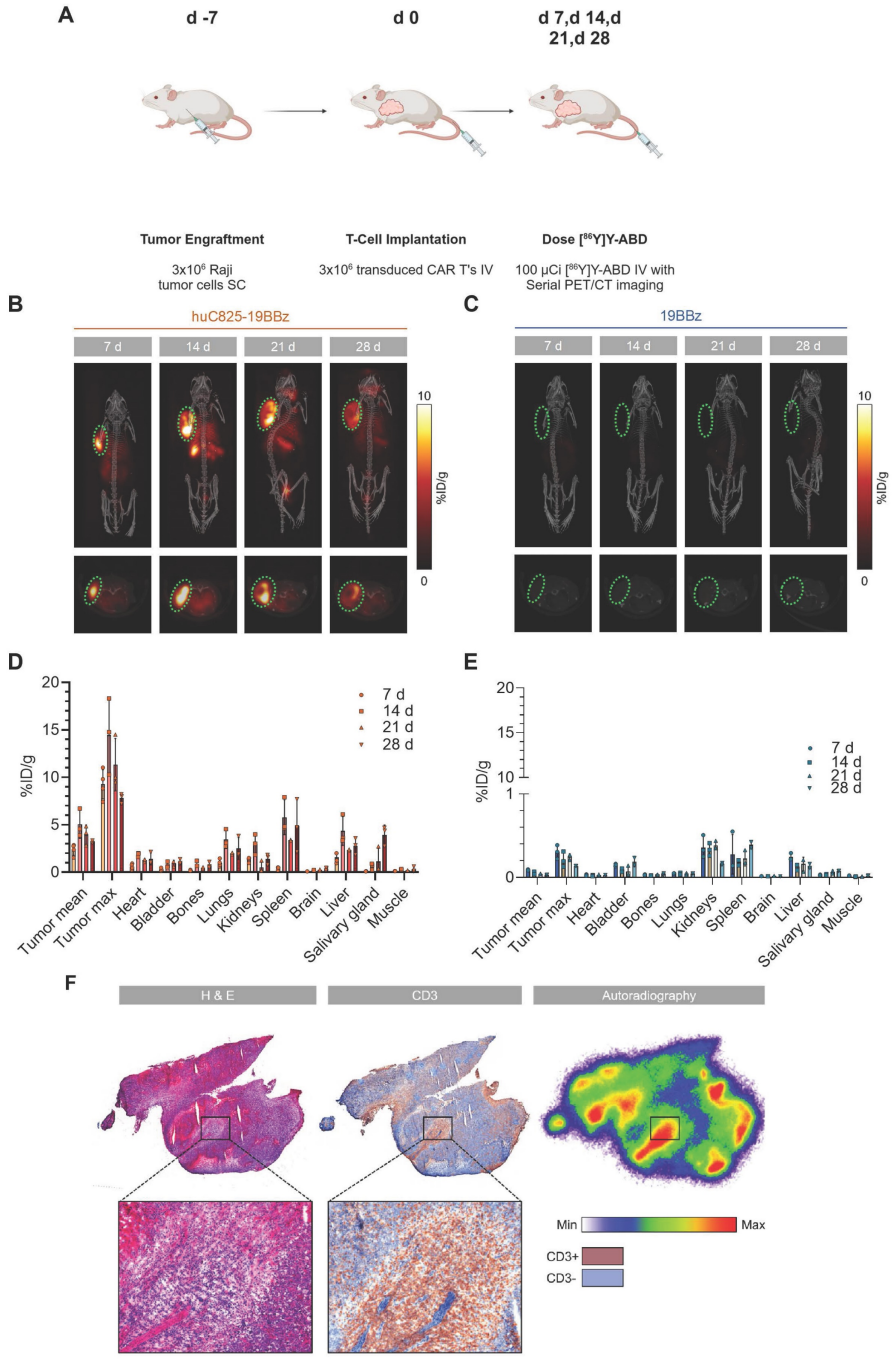
** [^86^Y]Y-ABD PET/CT visualizes huC825-19BBz T cell trafficking to the tumor and normal tissues with exceptionally high contrast over extended periods of time.** (A) Schema. Raji cells (3x10^6^) were implanted subcutaneously into NSG mice, injected with huC825-CAR or CAR-T cells (3x10^6^) intravenously seven days later, and weekly imaged by PET/CT after intravenous radiotracer ([^86^Y]Y-ABD) injection. (B, C) Maximum intensity projection (MIP) and axial PET/CT images at d 7, d 14, d 21 and d 28. In mice transfused with huC825-19BBz T cells, intense uptake at the tumor site (circle, red) already at d 7, as well as uptake in normal tissues, such as lungs, and spleen. No uptake is seen in mice injected with 19BBz T cells (tumor marked by circle, blue). (D, E) Image-based biodistribution. For tumor [%ID/g]_max_ and [%ID/g]_mean_ and for normal tissues [%ID/g]_mean_ are provided. (mean ± SD, n = 3, *n = 2) (F) Autoradiography and immunohistochemistry of tumor tissue containing huC825-19BBz T cells confirm “homing.” Radiotracer co-localizes with CD3-positive T cell cluster (brown staining). H&E = hematoxylin and eosin.

To investigate huC825 reporter-based CAR-T cell tracking using PET/CT *in vivo*, we injected 3×10^6^ Raji tumor cells (s.c.) on the shoulder of NSG mice on day -7, followed by 3×10^6^ 19BBz or Thor-cells (i.v.) on day 0. On day 7, 14, 21, and 28 post-T-cell injection, animals were intravenously injected with [^86^Y]Y-ABD (3.7 MBq) (Figure S4) and whole-body PET/CT imaging was performed (Figure 2A). We observed tumor-associated heterogenous uptake in mice injected with Thor-cells as early as day 7 post-T-cell administration (8.4 ± 2.1 %ID/g_max_, n = 3), which peaked at day 14 (14.2 ± 2.9 %ID/g_max_, n = 3) and persisted until the last imaging timepoint (day 28 post-T-cell injection) (Figure 2B, C). Similarly, we observed an increase in the Thor-cell to tumor cell ratio by *ex-vivo* flow cytometry until day 14 (Figure S5). No differences were observed in the anti-tumor effects of Thor-cells and 19BBz T cells (Figure S6). Furthermore, uptake in other tissues such as the lungs and spleen, and at later timepoints in the salivary gland, was noted. In contrast, minimal uptake at the tumor site (0.090 ± 0.007 %ID/g, n = 4, day 7 post-T-cell injection) and normal tissues was seen in control mice receiving 19BBz or huC825-GFP T cells (Figure 2C-E, Figure S7-S8). We confirmed the presence of Thor-cells in tumor samples harvested from mice at day 28 post-T-cell injection; autoradiography and CD3 staining of tumor tissue showed that the areas with the highest activity concentration were those infiltrated by Thor-cells (Figure 2F). A total of 13,350,000 T cells were present in the tumor tissue (Figure S9). Furthermore, autoradiography and CD3 staining of salivary gland tissue confirmed that the radioactivity localized to infiltrated Thor-cells (Figure S10), thus confirming off-site CAR-T localization. These data demonstrate that Thor-cells can be monitored *in vivo* over extended periods of time and the signal observed via PET/CT is specific, related to radiohapten capture by Thor-cells. The huC825 platform also uncovered Thor-cell localization to off-target tissues.

### Thor-cells can be characterized on a tissue level

**Figure 3:**
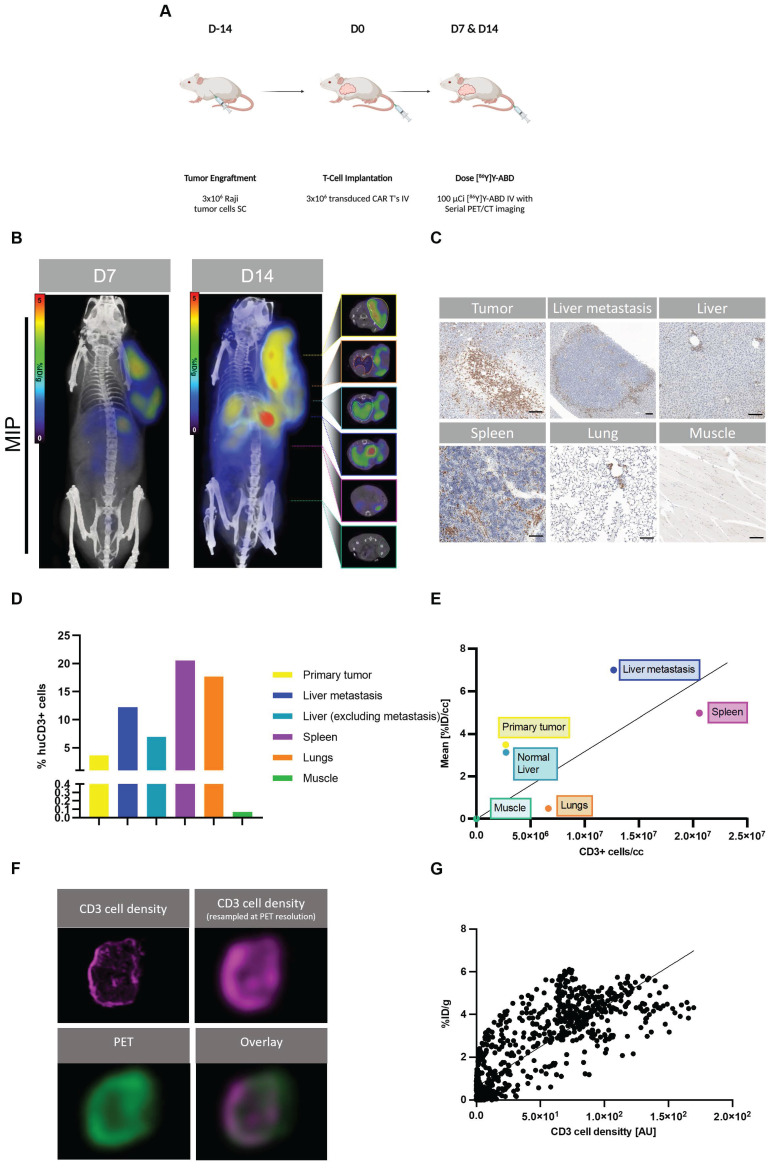
** Thor-cells can be characterized on a tissue level.** (A) Raji cells (3x10^6^) were implanted subcutaneously into NSG mice, injected with CAR-T cells (3x10^6^) intravenously 14 days later and imaged with PET/CT 16 h post intravenous radiotracer ([^86^Y]Y-ABD) injection (3.7 MBq). (B) Serial PET/CT imaging in Raji-bearing NSG mice after administration of huC825-19BBz. Maximum intensity projection (MIP) and axial images of representative mouse shown at d 7 and d 14 (n = 4). (C) IHC of CD3 (T cells) in selected tissues excised after the last imaging timepoint at d 14 post-injection of Thor-cells. Scale bar= 100 µm (D) Quantification of CD3^+^ cell population. (E) Correlation of CD3^+^ cells/section and respective radiotracer uptake on PET/CT [%ID/g]. (R^2^=0.23, p > 0.1 (F) Pixel by pixel overlay of CD3^+^ cell density map and PET image of tumor tissue. (G) Correlation of the CD3/PET pixel overlay, CD3 cell density in [AU], Pearson correlation, R^2^=0.7, p < 0.001.

Next, we aimed to investigate the correlation of uptake on PET with the Thor-cell biodistribution on a tissue level in a larger tumor burden mouse model. We injected 3x10^6^ Raji tumor cells (s.c.) into the shoulder of NSG mice on day -14, followed by 3x10^6^ Thor-cells (i.v.) on day 0. On days 7 and 14 post-CAR injection, animals were intravenously injected with [^86^Y]Y-ABD (3.7 MBq) and whole-body PET/CT imaging was performed, followed by *ex vivo* immunohistochemical analyses of selected organs (Figure 3A). PET/CT imaging showed heterogenous radiotracer at the primary tumor site on day 7 (5.7 ± 2.0 %ID/g_max_, n = 3), similarly at day 14 (5.2 ± 1.0 %ID/g_max_, n = 3) (Figure 3B, Figure S11), with increasing uptake in normal tissues, such as the lungs and spleen.

Interestingly, these mice with a primary tumor in an advanced stage showed focal increased radiotracer uptake in the liver at day 14 that was even higher than at the primary tumor site (8.8 ± 1.8 %ID/g_max_, n = 3) (Figure 3B, Figure S11). The uptake on PET correlated with sites of secondary tumor growth on immunohistochemistry, including a distant lymphoma in the liver of 1.5 mm diameter (Figure 3C-D, Table S2). CD3 staining confirmed densely clustered Thor-cells surrounding the secondary tumor site in the liver tissue. Diffuse uptake in the remainder of the liver was seen, corresponding to limited Thor-cell infiltrates. Interestingly, in the representative mouse shown, the spleen contained high levels of CD3^+^ cells (20600 CD3^+^ cells/mm^3^) associated with rather low uptake on PET (4.3 %ID/g_mean_), while the liver-derived distant lymphoma had fewer CD3^+^ cells (12700 CD3^+^ cells/mm^3^), but markedly higher uptake (6.5 %ID/g_mean_) (Figure 3B-E, Table S2). In the lungs, notable accumulation of Thor-cells within the alveolar septa (intravascular), and moderate multifocal perivascular (extravascular) infiltrates were observed (Figure 3C-D, Table S2). The pattern of infiltration in the normal lung and liver tissues is very common in immunodeficient mice that have been administered human T cells 16.

Next, we investigated the correlation between the enumerated CD3^+^ cells and the radiotracer uptake on PET; potential intratumoral (i.e., pixel-by-pixel) and inter-organ correlations were considered. We observed only a weak correlation between the mean number density of CD3^+^ cells in selected organs/tumors and corresponding mean [^86^Y]Y-ABD uptake (R^2^=0.23, p > 0.1) (Figure 3E, Table S2). However, PET and IHC signals correlated well on a pixel-by-pixel basis (R^2^=0.70, p < 0.0001) (Figure 3F-G) when the CD3 cell density of the primary tumor tissue was resampled at PET resolution.

Taken together, these data strongly support the hypothesis that [^86^Y]Y-ABD PET/CT effectively tracks and is specific to Thor-cell infiltration and thus further supports its pharmacologic utility.

**Figure 4:**
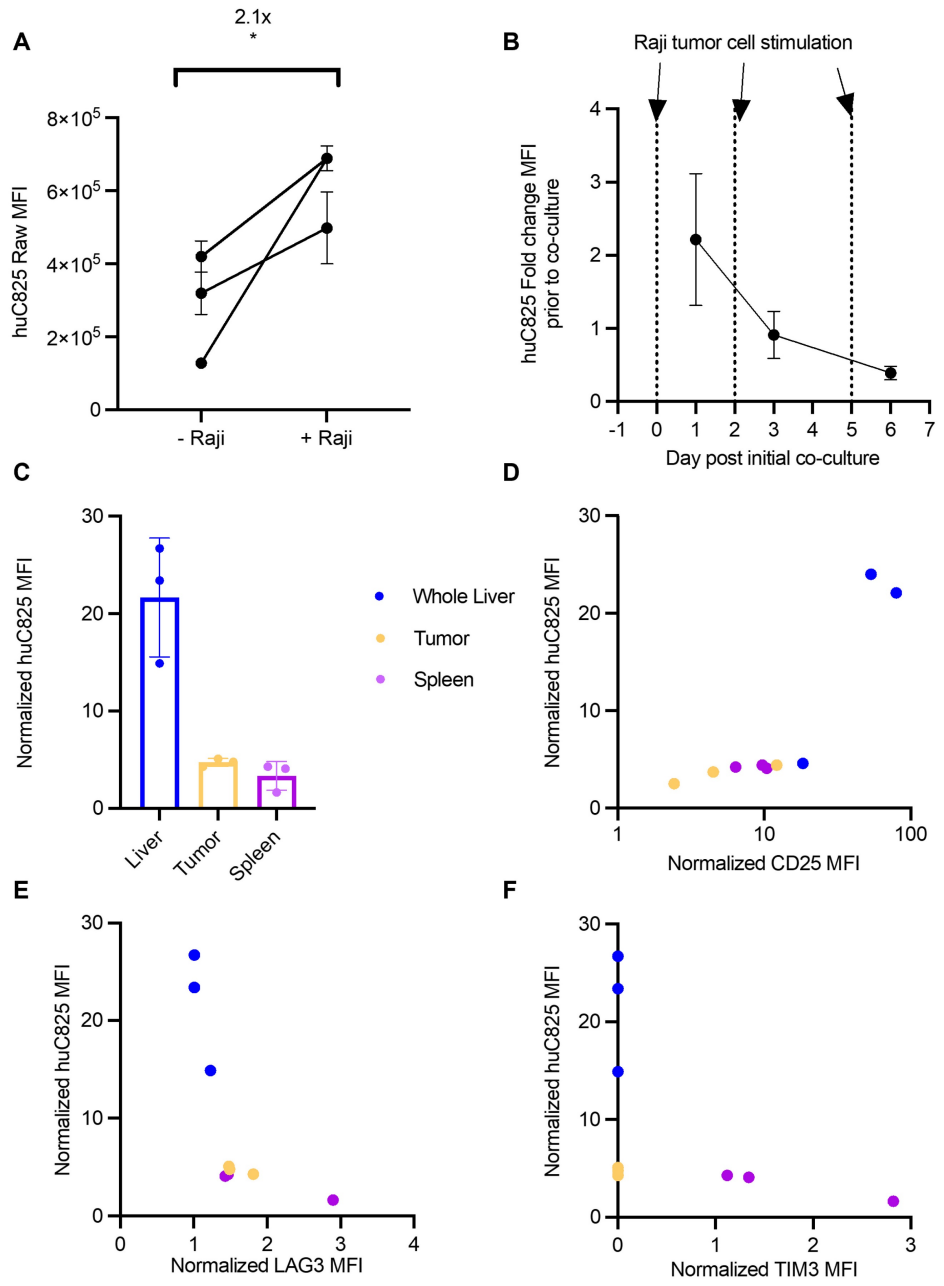
** Thor-cells' huC825 expression is dependent upon their activation and exhaustion status.** (A) Raji cells and huC825-19BBz cells were co-cultured at a 1:1 ratio (1x10^5^ total cells) and huC825 levels were measured using flow cytometry with [Biotin]-Pr 16 h post co-culture. (*: p < 0.05, unpaired, two-tailed t-test). (B) Co-culture was repeated as in (A) with additional Raji cells added at d 2 and d 5. huC825 fold change MFI was normalized to the huC825 MFI from untreated huC825-19BBz cells on d 0. (C) Harvested tissues normalized huC825 MFI is depicted for mice treated as in Figure 3A. (D, E, F) Normalized CD25, LAG3 and TIM3 MFI is correlated to huC825 MFI. (Spearman's, CD25: R^2^=0.921, p = 0.001, LAG3: R^2^= -0.75623, p = 0.0228, TIM3: R^2^=-0.9528, p = 0.0002). Normalized MFI's for (C-F) were calculated as fold change over the FMO. 3 representative donors were utilized for (A-B).

In view of the discrepancy in correlation of uptake on PET and CD3 staining in normal organs and tumor sites versus at the tissue level, we tested whether antigen stimulation alters the expression of huC825 on Thor-cells, thus contributing to the differential radiotracer uptake observed in these tissues. We demonstrated by flow cytometry that Thor-cells co-cultured with Raji cells *in vitro* have increased binding capacity for Biotin-Pr, a probe specific for huC825 expression, compared to unstimulated Thor-cells (Figure 4A). To further understand huC825 kinetics, we repeatedly stimulated Thor-cells with Raji cells *in vitro*; we observed stimulation-dependent reduction in huC825 expression (d1=2.2-, d3=0.9-, d6=0.39- fold change, relative to unstimulated Thor-cells) (Figure 4B).

Since the above data indicated that huC825 is dependent on antigen stimulation, we hypothesized that the functional state of Thor-cells could explain the differences in radiotracer uptake between the liver-derived distant lymphoma and other tissues, such as the spleen. We performed flow cytometry on tumor, spleen, and liver samples harvested 14 days after T cell injection (as in Figure 3). Thor-cells located in the liver had 5- and 7-fold higher levels of huC825-expression relative to the primary tumor and spleen, respectively (Figure 4C, Figure S12), thus strongly suggesting that the focal intense liver uptake we observed by PET/CT is likely due to increased huC825 expression by Thor-cells associated with the liver-derived distant lymphoma. In these three tissues we also found that there was positive correlation between the amount of CD25, a T cell activation marker, and huC825 (R^2^=0.921, p = 0.001, Figure 4D, Figure S12). Conversely, we show negative correlation of LAG3 and TIM3 exhaustion marker expression with huC825 expression across all three tissue types (LAG3: R^2^=-0.75623, p = 0.0228; TIM3: R^2^=-0.9528, p = 0.0002; Figure 4E-F, Figure S12). We also confirmed this correlation pattern with the percent of gated huC825 cells with each of these markers (Figure S13A-C). Additionally, the MFI of the 19E3 CAR correlates with TIM3 and LAG3 MFI (Figure S13D-E), thus confirming the exhaustion patterns of these cells. This dependence of radiotracer uptake on T cell activation and exhaustion suggests the Thor system enables preferential tracking of the most functional T cells.

### Radiotherapeutic engaged Thor-cells kill tumor in an orthogonal manner to their intrinsic T cell function

**Figure 5:**
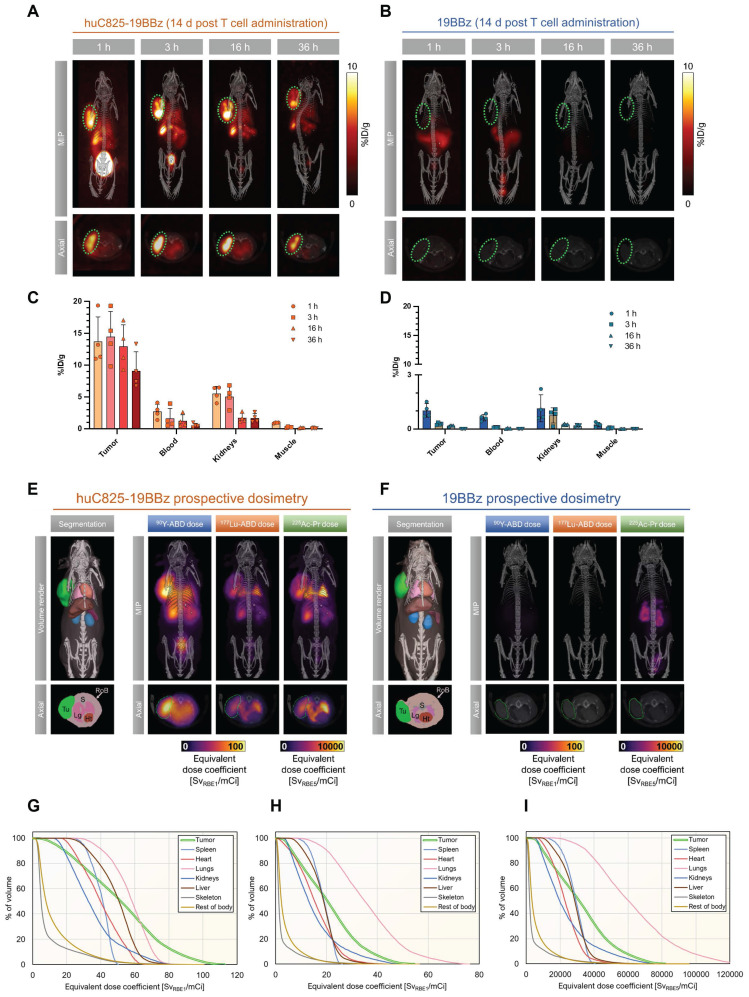
** Image-based dosimetry for therapeutic analogs of [^86^Y]Y-ABD: [^177^Lu]Lu-ABD, [^90^Y]Y-ABD, and [^225^Ac]Ac-Pr.** (A, B) Raji cells (3x10^6^) were implanted subcutaneously into NSG mice, injected with huC825-19BBz or 19BBz T cells (3x10^6^) intravenously seven days later and imaged by PET/CT on d 14 at 1, 3, 16 and 36 h post intravenous radiotracer ([^86^Y]Y-ABD) (3.7 MBq) injection. Maximum intensity projection (MIP) and axial images of representative mice shown. n = 3 (C,D) Image-based biodistribution. For tumor [%ID/g]_max_ and for normal tissues [%ID/g]_mean_ are provided. (mean ± SD, n = 3) (E,F) MOBY mouse phantom used in Monte Carlo absorbed dose calculations. Absorbed dose maps (maximum intensity projections) for [^90^Y]Y-ABD, [^225^Ac]Ac-Pr and [^177^Lu]Lu-ABD estimated prospectively from [^86^Y]Y-ABD biodistribution measurements (G,H,I) Organ-level mean absorbed doses from calculations in (E) for [^90^Y]Y-ABD (G), [^177^Lu]Lu-ABD (H), and [^225^Ac]Ac-Pr (I).

CAR-T therapy may fail to exhibit successful anti-tumor effects. DOTA-haptens, including our next generation “Proteus” DOTA-platform (Pr), can conveniently be labeled with a variety of radionuclides for imaging or therapeutic approaches (Figure S4). To this end, we investigated [^86^Y]Y-ABD as a theranostic surrogate for augmentation of Thor-cell killing capacity by *in vivo* labeling with the therapeutic analogs, [^177^Lu]Lu-ABD, [^90^Y]Y-ABD and [^225^Ac]Ac-Pr in the same Raji xenograft model (*vide supra,* Figure 2A). Using image-based organ biodistribution, we calculated murine and human dose estimates for [^86^Y]Y-ABD and prospective dosimetry for the therapeutic haptens via quantification of [^86^Y]Y-ABD at 1, 3, 16, and 36 h post-tracer administration in mice injected with Thor-cells 14 days prior (Figure 5A-D, Figure S14, Table S3).

Favorable biodistribution with rapid clearance through the kidneys was noted, resulting in exceptionally high contrast in PET imaging (Figure S15). Normal organ dose estimates were favorable for repeated PET/CT studies in both mice and humans (Tables S3, S4). Monte Carlo radiation dose modeling demonstrated prospective maximal lesion equivalent dose coefficients exceeding 110 Sv/mCi (RBE 1) for [^90^Y]Y-ABD therapy, exceeding 50 Sv/mCi (RBE 1) for [^177^Lu]Lu-ABD therapy and exceeding 83,000 Sv/mCi (RBE 5) for [^225^Ac]Ac-Pr therapy. The mean tumor equivalent dose coefficients were 52 and 21 Sv/mCi, respectively, for [^90^Y]Y-ABD and [^177^Lu]Lu-ABD, and 33,000 Sv/mCi for [^225^Ac]Ac-Pr. The critical organ in all cases was the lung, which received 58 Sv/mCi for [^90^Y]Y-ABD, 37 Sv/mCi for [^177^Lu]Lu-ABD and 65,000 Sv/mCi for [^225^Ac]Ac-Pr. The dose estimates for normal tissues were approximately 1-2 orders of magnitude higher for mice administered huC825 CAR-T cells vs. CAR-T cells (Figure 5E-F, Table S4), indicating the T cell distribution is a principal driver of normal tissue radiation dose in this model. Thus, prospective dose estimates for the therapeutic radiohaptens suggested the feasibility of enhancement of overall therapeutic potential of Thor-cells, but also identified the lung as the dose-limiting organ at this timepoint, related to the T cells biodistribution (Figure 5E-I and Table S3). Given this information, the optimal timing for radiotherapeutic delivery is 7 days post-CAR-T administration based on three factors 1) peak therapeutic index based on PET and biodistribution 2) T cells activation and peak huC825 expression and 3) T cells are in expansion phase.

We selected [^225^Ac]Ac-Pr for characterization in the following efficacy model because of its short range of emission coupled with high potency (high LET) to minimize damage to the peripheral organs harboring Thor-cells. We confirmed a lack of systemic toxicity induced by [^225^Ac]Ac-Pr, as measured by mouse weight, blood cell counts and pathologic assessment in non-tumor-bearing NSG mice at administered doses up to 148 kBq (4 µCi) [^225^Ac]Ac-Pr with non-significant short-term leukopenia or erythropenia in both groups (Figure S16, Table S5).

We injected 3×10^6^ Raji tumor cells (s.c.) into NSG mice (day -7), followed by 3x10^6^ control 19BBz T or Thor-cells (i.v.) on day 0, and 74 kBq (2 µCi) of [^225^Ac]Ac-Pr (i.v.) on day 7 (Figure S17A). In this sub-therapeutic CAR-T model, we observed a slight decrease in relative bioluminescence-measured tumor burden of mice receiving Thor + [^225^Ac]Ac-Pr versus Thor-cells alone, or those receiving 19BBz ± [^225^Ac]Ac-Pr (Figure S17B). As measured by calipers, tumor regression was more pronounced in the Thor + [^225^Ac]Ac-Pr group (599 mm^3^) compared to Thor alone (1921 mm^3^) (Figure S17C). Importantly, we also observed a significant survival benefit in Thor + [^225^Ac]Ac-Pr-treated mice (median survival/MS = 32 days) versus Thor alone across two independent experiments (Figure S17D, MS = 26 days; p = 0.0357, n = 6). There was no statistical difference in median survival between NSG mice treated with 19BBz T cells ± [^225^Ac]Ac-Pr (Figure S17E). Additionally, we observed no therapeutic benefit of systemic delivery of [^225^Ac]Ac-Pr *in vivo*, thus confirming localized delivery of this therapeutic payload by the Thor-cell is required (Figure S18).

Damage to the Thor-cell itself by a highly energetic payload such as Ac-225, is a major concern, so we investigated [^225^Ac]Ac-Pr mediated depletion of Thor-cells *in vivo*. Tumor samples from mice treated with [^225^Ac]Ac-Pr showed decreased numbers of Thor-cells compared to the control group (Figure S17F). However, [^225^Ac]Ac-Pr does not completely eliminate Thor-cells, which suggests the potential for repeat therapeutic radiohapten dosing to increase the anti-tumor effect. These data demonstrate that Thor-cell therapy may be augmented by systemically administered therapeutic DOTA-haptens with minimal risk of systemic toxicities.

## Discussion

This study presents a novel radiohapten capture platform enabling monitoring of tumor-targeted T cells *in vivo* using PET, as well as providing selective delivery of therapeutic radioisotopes to increase anti-tumor efficacy. To our knowledge, we are the first to report this combinatorial strategy of real-time CAR-T cell monitoring and localized delivery of therapeutic radionuclide within tumors by CAR-T cells, and thus represents a major advancement for CAR-T cell therapy.

The Thor platform includes a DOTA-based reporter probe, a chelate that is commonly employed as part of MRI contrast agents and radiopharmaceuticals in the clinic. We demonstrated that the reporter huC825 can be expressed on lymphocytes and CAR-T cells without altering effector functions *in vitro* and *in vivo*. The picomolar affinity of huC825 to metalated DOTA, in conjunction with the rapid, predominantly renal clearance of unbound DOTA-haptens, facilitates detection of Thor-cells at least as early as 1 h post-injection of the radiotracer, which continues to increase at later timepoints. Indeed, at 16 h post-radiotracer administration, all unbound radiotracer has cleared, while [^86^Y]Y-ABD captured by the engineered T cells remained bound, thus resulting in excellent T cell-to-background ratios and high-contrast images. These characteristics also resulted in high levels of sensitivity, as we detected approximately 20,000 Thor-cells at small sites of distant lymphomatous disease.

Especially in view of the challenge of detecting low amounts of T cells accumulating at tumor deposits or normal tissues in mouse models and in the clinical setting, high image contrast and lack of non-specific radiotracer uptake are requisites for a clinically relevant reporter system. Non-specific tracer uptake has been observed in a clinical study using HSV1-tk reporter and the probe [^18^F]-FHBG to track CAR-T cells targeting interleukin-13 receptor alpha 2, necessitating the performance of a baseline scan prior to T cell administration and subsequent follow up reporter-based imaging 10. Furthermore, in human reporter gene systems, such as SSTR2 or PSMA, target expression is not restricted to the engineered T cells, and tracer uptake in normal tissues, tumor tissues or tumor microenvironment expressing the target is seen, thus limiting specificity of uptake. In contrast, the DOTA-reporter gene/probe system in which expression is confined to the engineered T cells avoids these confounding factors. Our observed detection sensitivity *in vivo* compares well with prior data using other reporter genes. Sakemura et el. reported a detection limit of 40,000 cells using the NIS reporter gene in conjunction with [^18^F]-TFB PET 17. CAR-T cells expressing SSTR2-reporter could be detected with a minimum density of 0.8% or ∼4 × 10^6^ cells/cm^3^ with 95% specificity and 87% sensitivity in tumor located in the lung using [^68^Ga]Ga-DOTATOC 13. PSMA-reporter bearing CAR-T cells could be detected as low as 2000 cells 11.

In our prior study using the murine DOTA-antibody reporter DAbR1 and the probe [^86^Y]Y-AABD for *in vivo* tracking of 3x10^6^ i.v. injected CD19 CAR-T cells in a subcutaneous high-burden CD19^+^ tumor model, we observed heterogeneously increased uptake at the tumor site below 1 %ID/g at 16 h post-tracer administration on day 10 post-T cell injection 14. Allowing for differences in the tumor model and generation of the engineered T cells, the substantial higher uptake observed with the huC825 reporter system is likely related to the differences in affinity between reporter and probe.

An ideal tool for monitoring CAR-T therapies enables tracking of the T cells' migration over extended periods of time, their engagement with the antigen-expressing tumor cells, their expansion and persistence, and their localization at off-tumor sites. These features are necessary for development of effective and safe cell therapies 9. We showed that Thor-cells can be serially imaged, localize at tumor sites and in normal tissue, and persist for several weeks. We observed a strong correlation of the uptake on PET with the distribution of the T cells on a tissue level. Intriguingly, the radiohapten capture on the cells is directly tied to huC825 expression, which is in turn regulated by CAR-T activation and exhaustion. Indeed, it has been described that retroviral vectors are expressed more by activated T cells 18, 19. Thus, the Thor platform enables preferential tracking of the most functional T cells. This platform might have the potential to predict the anti-tumor activity of CAR-T therapies and off-tumor CAR-T-mediated toxicities, in addition to its utility as a pharmacokinetic tool. Indeed, we uncovered off-tumor localization in the salivary gland, which suggested the possible onset of graft versus host disease. The potential to monitor off-tumor toxicities is a particularly important feature to support IND applications of novel specificity CARs or other newly engineered cellular platforms.

Furthermore, we report the first demonstration of augmented anti-tumor efficacy of a CAR-T cell therapy with a cytotoxic α-emitting payload by utilizing the CAR-T cells themselves as the *in situ* radioactive drug localization tool. The Thor concept represents an important embodiment of targeted 'micropharmacies,' a novel strategy of CAR-T enhancement recently proposed by our group 20, 21. We also show that prospective dosimetry can identify organs at risk for side effects from α- and β-radionuclide therapies, underscoring the benefit of a dosimetry-based treatment approach. Moreover, the human dosimetry estimate for [^86^Y]Y-ABD is comparable to other radiometallated small molecule PET agents and suggests that [^86^Y]Y-ABD can be safely administered to patients for single or serial imaging studies.

In summary, the Thor-cell theranostic approach is a combination of a sensitive and specific methodology for tracking the pharmacokinetics of CAR-T cells *in vivo* as well as a therapeutic strategy that has the potential to enhance CAR-T cell efficacy with α-emitting radiohaptens. This theranostic strategy may aid in the development of future CAR-T cell therapies and improve clinical outcomes in patients.

## Conclusion

We present a novel modular radiohapten capture platform that enables serial real-time, whole-body monitoring of CAR-T cells' pharmacokinetics using PET as well as selective delivery of cytotoxic radioisotopes into tumors. Thus, Thor-cells are a new theranostic agent that may enable early prediction of treatment response, early recognition of off-site targeting, facilitate optimizing treatment approaches and translation of novel specificity CAR-T cells. The delivery of therapeutic radionuclides may improve CAR-T treatment efficacy including in CAR antigen-loss variants.

## Supplementary Material

Supplementary materials and methods, figures and tables.

## Abbreviations

CAR: chimeric antigen receptor
DOTA: 1,4,7,10-tetraazacyclododecane-1,4,7,10-tetraacetic acid
scFv: single-chain variable fragment
PET/CT: positron emission tomography/computerized tomography

## References

[1] Brentjens RJ, Davila ML, Riviere I, Park J, Wang X, Cowell LG (2013). CD19-targeted T cells rapidly induce molecular remissions in adults with chemotherapy-refractory acute lymphoblastic leukemia. Sci Transl Med.

[2] Kochenderfer JN, Dudley ME, Kassim SH, Somerville RP, Carpenter RO, Stetler-Stevenson M (2015). Chemotherapy-refractory diffuse large B-cell lymphoma and indolent B-cell malignancies can be effectively treated with autologous T cells expressing an anti-CD19 chimeric antigen receptor. J Clin Oncol.

[3] Maude SL, Laetsch TW, Buechner J, Rives S, Boyer M, Bittencourt H (2018). Tisagenlecleucel in children and young adults with B-cell lymphoblastic leukemia. N Engl J Med.

[4] Krebs S, Rodriguez-Cruz TG, Derenzo C, Gottschalk S (2013). Genetically modified T cells to target glioblastoma. Front Oncol.

[5] Krebs S, Chow KK, Yi Z, Rodriguez-Cruz T, Hegde M, Gerken C (2014). T cells redirected to interleukin-13Ralpha2 with interleukin-13 mutein-chimeric antigen receptors have anti-glioma activity but also recognize interleukin-13Ralpha1. Cytotherapy.

[6] Hegde M, Mukherjee M, Grada Z, Pignata A, Landi D, Navai SA (2016). Tandem CAR T cells targeting HER2 and IL13Ralpha2 mitigate tumor antigen escape. J Clin Invest.

[7] Flugel CL, Majzner RG, Krenciute G, Dotti G, Riddell SR, Wagner DL (2023). Overcoming on-target, off-tumour toxicity of CAR T cell therapy for solid tumours. Nat Rev Clin Oncol.

[8] Krebs S, Dacek MM, Carter LM, Scheinberg DA, Larson SM (2020). CAR chase: where do engineered cells go in humans?. Front Oncol.

[9] Krebs S, Ponomarev V, Slovin S, Schöder H (2019). Imaging of CAR T-cells in cancer patients: paving the way to treatment monitoring and outcome prediction. J Nucl Med.

[10] Keu KV, Witney TH, Yaghoubi S, Rosenberg J, Kurien A, Magnusson R (2017). Reporter gene imaging of targeted T cell immunotherapy in recurrent glioma. Sci Transl Med.

[11] Minn I, Huss DJ, Ahn HH, Chinn TM, Park A, Jones J (2019). Imaging CAR T cell therapy with PSMA-targeted positron emission tomography. Sci Adv.

[12] Zhang H, Moroz MA, Serganova I, Ku T, Huang R, Vider J (2011). Imaging expression of the human somatostatin receptor subtype-2 reporter gene with ^68^Ga-DOTATOC. J Nucl Med.

[13] Vedvyas Y, Shevlin E, Zaman M, Min IM, Amor-Coarasa A, Park S (2016). Longitudinal PET imaging demonstrates biphasic CAR T cell responses in survivors. JCI Insight.

[14] Krebs S, Ahad A, Carter LM, Eyquem J, Brand C, Bell M (2018). Antibody with Infinite Affinity for In Vivo Tracking of Genetically Engineered Lymphocytes. J Nucl Med.

[15] Dacek MM, Veach DR, Cheal SM, Carter LM, McDevitt MR, Punzalan B (2021). Engineered cells as a test platform for radiohaptens in pretargeted imaging and radioimmunotherapy applications. Bioconjug Chem.

[16] Curran M, Mairesse M, Matas-Céspedes A, Bareham B, Pellegrini G, Liaunardy A (2020). Recent advancements and applications of human immune system mice in preclinical immuno-oncology. Toxicol Pathol.

[17] Sakemura R, Bansal A, Siegler EL, Hefazi M, Yang N, Khadka RH (2021). Development of a clinically relevant reporter for chimeric antigen receptor T-cell expansion, trafficking, and toxicity. Cancer Immunol Res.

[18] Lin HC, Hickey M, Hsu L, Medina D, Rabson AB (2005). Activation of human T cell leukemia virus type 1 LTR promoter and cellular promoter elements by T cell receptor signaling and HTLV-1 Tax expression. Virology (Lond).

[19] Burns WR, Zheng Z, Rosenberg SA, Morgan RA (2009). Lack of specific gamma-retroviral vector long terminal repeat promoter silencing in patients receiving genetically engineered lymphocytes and activation upon lymphocyte restimulation. Blood.

[20] Gardner TJ, Lee JP, Bourne CM, Wijewarnasuriya D, Kinarivala N, Kurtz KG (2022). Engineering CAR-T cells to activate small-molecule drugs in situ. Nat Chem Biol.

[21] Gardner TJ, Bourne CM, Dacek MM, Kurtz K, Malviya M, Peraro L (2020). Targeted cellular micropharmacies: cells engineered for localized drug delivery. Cancers (Basel).

