# Supplementary material for: Engineering CAR-T cells for radiohapten capture in imaging and radioimmunotherapy applications
Source: Theranostics. 2023 Oct 8;13(15):5469–82. doi: 10.7150/thno.87489 (PMC10614694; doi:10.7150/thno.87489)
Supplement: Supplementary file 1 — Supplementary materials and methods, figures and tables. [file thnov13p5469s1.pdf]

Supplemental Material for:

Engineering CAR-T cells for radiohapten capture in imaging and radioimmunotherapy applications

Keifer Kurtz[1,2#], Laura Eibler[3#], Megan M. Dacek[1,2], Lukas M. Carter[4], Darren R. Veach[5,6], Samantha Lovibond[3], Emma Reynaud[3], Sarah Qureshy[1], Michael R. McDevitt[5,6], Christopher Bourne[1,7], Sebastien Monette[8], Blesida Punzalan[1], Shireen Khayat[1,2], Svena Verma[2], Adam L. Kesner[4], Nai-Kong V. Cheung[9], Heiko Schöder[3,6], Leah Gajecki[3], Sarah M. Cheal[1,6], Steven M. Larson[1,3,6##], David A. Scheinberg[1,2##], Simone Krebs[3,5,6##,*]

[1]Molecular Pharmacology Program, Sloan Kettering Institute, Memorial Sloan Kettering Cancer Center, New York, NY 10065, USA

[2]Department of Pharmacology, Weill Cornell Medical College, New York, NY 10065, USA

[3]Molecular Imaging and Therapy Service, Department of Radiology, Memorial Sloan Kettering Cancer Center, New York, NY 10065, USA

[4]Department of Medical Physics, Memorial Sloan Kettering Cancer Center, New York, NY 10065, USA

[5]Radiochemistry and Imaging Sciences Service, Department of Radiology, Memorial Sloan Kettering Cancer Center, New York, NY 10065, USA

[6]Department of Radiology, Weill Cornell Medical College, New York, NY 10065, USA

[7]Immunology and Microbial Pathogenesis Program, Weill Cornell Medical College, New York, NY 10065, USA

[8]Laboratory of Comparative Pathology, Memorial Sloan Kettering Cancer Center, Weill Cornell Medicine, and The Rockefeller University, New York, NY 10065, USA

[9]Department of Pediatrics, Memorial Sloan Kettering Cancer Center, New York, NY 10065, USA

***Corresponding Author**:

Simone Krebs, Memorial Sloan Kettering Cancer Center, 1275 York Ave, Box 77, New York, NY 10065; Email: krebss@mskcc.org, Phone: 212-639-7373, Fax: 212-717-3263

[#]Contributed equally as first authors

[##]Contributed equally as senior authors

Table of Contents:

1. **Methods and Materials**
2. **Tables S1-S5**
3. **Figures S1-S18**
4. **References**

Methods

Assembly of huC825 Reporter Gene

A geneblock encoding the membrane bound huC825s was purchased (Integrated DNA Technologies). The geneblock consists of the human CD4 endoplasmic reticulum signal sequence, the scFv huC825, and a CD4 transmembrane domain. Gibson assembly was used to clone the huC825s sequence into an SFG retroviral vector harboring the DNA sequence for the anti-CD19 19BBz CAR (huC825s-19BBz). The IgG4-CH2CH3 spacer domain with mutated Fc regions (hinge) was added to the geneblock using Gibson assembly between the scFv huC825 and the CD4 transmembrane domain; this sequence was also cloned into the 19BBz viral vector to generate huC825-19BBz. huC825-GFP was also generated by Gibson assembly of the huC825 sequence into a retroviral vector encoding eGFP. 19BBz SFG retrovirus was generously provided by the Renier Brentjens lab (Memorial Sloan Kettering Cancer Center/MSK). Retroviral sequences were verified by sequence analysis (Eton Bioscience).

Cell Culture

All cell lines and human PBMC's were grown in RPMI-1640 medium (MSK Center Media Core). All media was supplemented with 10% fetal bovine serum, 2 mMol L-glutamine, 100 IU/mL penicillin and 100 µg/mL streptomycin. Raji tumor cells were purchased from ATCC and transduced to express eGFP and firefly luciferase (Raji fluc/eGFP cells) using viral supernatant

from Galv9 cells producing SFG retrovirus encoding the DNA for each of these proteins. All cell lines were regularly tested to ensure they were free of mycoplasma contamination.

Synthesis and Radiolabeling of [^{86}Y]Y-ABD, [^{111}In]In-Pr, and [^{225}Ac]Ac-Pr

Radiochemistry was performed in appropriately shielded chemical fume hoods equipped with electronic flow monitoring and sliding leaded glass windows. A CRC-55tR dose calibrator was used to measure radioactivity using manufacturer-recommended calibration settings (Capintec Inc., Florham Park,NJ). Buffers and water used for radiochemical synthesis were treated with 5% (w/v) Chelex ion exchange resin (BT Chelex 100 Resin, Bio-Rad Inc., Hercules, CA) to remove adventitious heavy metals, or were tracemetal grade. Plasticware (such as pipette tips and microcentrifuge tubes) was trace metal grade or PCR grade. Radio-HPLC was performed on a Shimadzu Prominence HPLC system (Shimazdu Scientific Instruments, Somerset, NJ) comprised of an LC-20AB dual pump module, DGU-20A3R degasser, SIL-20ACHT autosampler, SPD-20A UV-Vis detector and a Bioscan Flow-Count B-FC-1000 with PMT/NaI radioactivity detector in-line. Separations were run on a 4.6 × 250 mm Gemini-NX C18 column (Phenomenex Inc., Torrance, CA). HPLC conditions were: mobile phase A, 10 mM pH 5 NH_4OAc; B, CH_3CN; 1.0 mL/min flow rate; λ = 254 nm; injection volume 5–20 µL; gradient 0% B to 40% B over 10 min. Samples of free radiometals, reaction mixtures, and purified products were diluted 1:5 in 5 mM DTPA or EDTA prior to analysis. With the exception of unsubstituted DOTA, multiple peaks may be observed due to diastereomeric / helical isomerism of radiometallated DOTA species, these isomers bind to the anti-[M]DOTA radiohapten capture platform (C825).

Radiosynthesis of [^{111}In]In-Pr

No-carrier-added [^{111}In]$InCl_3$ (47.4 MBq/1.28 mCi) in 50 µL of 0.05 M HCl (Nuclear Diagnostic Products Inc., Plainview, NY) was transferred to a metal-free 0.5 mL microcentrifuge tube, diluted with 50 µL of metal-free 0.5 M NH_4OAc (pH 5.3), and mixed gently. To this was added 2.6 nmol

of proteus-DOTA (Pr; DO3A-PEG$_4$-[^{nat}Lu]Lu-DOTA-Bn; 2.6μL of 1mM solution in water) mixed gently by pipetting and placed in a heat block at 80 °C for 60 min. After cooling for 5 min, the entirety was gravity-loaded on a 30 mg Strata-X SPE cartridge (Phenomenex, Torrance, CA) that had been equilibrated with 1 mL of ethanol and 1 mL of water. Water (100 μL) was used to rinse the reaction tube and passed through the cartridge. The column was washed slowly dropwise with 200 μL of water and gently blown dry with nitrogen gas. The product was slowly eluted dropwise with 200 μL of ethanol, diluted as necessary in normal saline (Hospira, Lake Forest, IL) and sterile filtered to obtain "[^{111}In]In-Pr" [^{111}In]InDO3A-PEG$_4$-[^{nat}Lu]LuDOTA-Bn (40.7 MBq, 1.10 mCi, 86% ndc yield, A_M = 18.5 MBq/nmol, 500 μCi/nmol) at time of synthesis. Radio-HPLC of crude and purified material confirmed that no free radiometal remained (major isomer t_R = 8.0 min, 97.5% radiochemical purity).

Radiosynthesis of [^{86}Y]Y-2-(4'-aminobenzyl)DOTA; [^{86}Y]Y-ABD

[^{86}Y]Y-ABD was prepared in a similar manner as previously described[1]. Briefly, [^{86}Y]YCl_3 (252 MBq/6.80 mCi, A_M≥ 92MBq (≥ 2.5 mCi/nmol) in 300 μL of 0.04M HCl (UWCL; University of Wisconsin Cyclotron Lab, Madison, WI) was transferred to a metal-free 0.5 mL microcentrifuge tube, diluted with 300 μL of metal-free 0.5 M NaOAc (pH 5.5), and mixed gently. To this, DOTA-Bn, 4'-aminobenzylDOTA (Macrocyclics, Inc., Plano, TX; 305 nmol, 10 μL of 20 mg/mL solution in water) was added, mixed gently, and placed in a heat block at 90 °C for 30 min. After cooling for 5 min, complete conversion was confirmed by radio-HPLC and the crude material was gravity-loaded on a 30 mg Strata-X SPE cartridge (Phenomenex, Torrance CA) that had been equilibrated with 1 mL of ethanol and 1 mL of water. The column was washed with 100 μL of water and gently blown dry with nitrogen gas. The product, [^{86}Y]Y-ABD, was slowly eluted dropwise with 200-400 μL of ethanol (221 MBq/5.98 mCi; 87.7% ndc yield), then formulated with normal saline (Hospira, Lake Forest, IL) and sterile filtered (99.4% radiochemical purity; minor isomer t_R = 7.1min, major isomer t_R = 7.4 min). The molar activity was A_M = 0.73 MBq/nmol

(19.6 μCi/nmol) at time of synthesis; however, because only radiometallated ABD is recognized by the scFv C825, so the effective molar activity A_M(eff) is radiometal governed *in vivo*, i.e., ≥ 92 MBq/nmol (2.5 mCi/nmol) at the time of synthesis based on a production specification of 0.37–3.7 GBq/nmol (10–100 mCi/nmol) at the end of bombardment[2].

Radiosynthesis of [^{225}Ac]Ac-Pr

Carrier-free ^{225}Ac (2.146 x 10^6 GBq/g [5.80 x 10^4 Ci/g]) was obtained from NIDC, Oak Ridge National Laboratory (Oak Ridge, TN) as a dried nitrate residue. The ^{225}Ac nitrate was dissolved in 0.1 M Optima grade hydrochloric acid for subsequent radiochemistry. Ac-225 activity measurements were made at secular equilibrium prior to labeling using a CRC-55tR radioisotope calibrator (Capintec Inc., Florham Park, NJ) set at 775, and the displayed activity value multiplied by 5; samples were positioned at the bottom and center of the well for measurement. Reactions were typically run at 10–200 μCi scale; a representative process is as follows: [^{225}Ac]$AcNO_3$ in 0.1 M HCl (20 μL, 2.442 MBq / 66.0 μCi) was neutralized with 100 μL of 3 M ammonium acetate solution and 15 μL of L-ascorbic acid (150 g/L) in a glass reaction vial. To this was added 100 μL of 10 mg/mL Proteus-DOTA (Pr) (1 mg; 0.74 μmoles). The pH was confirmed (~5.5) by spotting 1 μL of the reaction mixture onto pHydrion pH paper (range 5.0–9.0). The reaction was incubated at 60 °C for 30 min and then purified using a CM Sephadex C-25 resin (Sigma-Aldrich) column pre-equilibrated with 6 mL of normal sterile isotonic saline solution (NSS). The reaction mixture was added to the column and eluted with 4 mL of NSS to recover all removable activity ([^{225}Ac]Ac-Pr); percentage of total activity eluted was used to determine yield and specific molar activity A_M. After formulation and sterile filtration, 2.29 MBq (62.0 μCi) of [^{225}Ac]Ac-Pr was obtained with a non-decay corrected radiochemical yield of 94% and A_M = 3.1 MBq/μmol (84 μCi/μmol). Good (>85%) yields were obtained up to A_M ≈ 120 MBq/μmol (3 μCi/nmol). Optimally, *in vivo* doses were formulated at 185–370 kBq/mL (5–10 μCi/mL) in saline

for a 200 µL injection volume. Doses for mice were typically <1 nmol and ~40–80 kBq (1–2 µCi) as specified elsewhere.

In vivo Experiments

All animal experiments were performed under a protocol approved by the Memorial Sloan Kettering Cancer Center Institutional Animal Care and Use Committee. For *in vivo* confirmation of huC825-19BBz anti-tumor activity and binding capacity for radiotracer, 5x10^5 Raji cells were implanted intraperitoneally (IP); two days later 5x10^5 T cells were also injected IP. On day 6, [^{86}Y]Y-ABD (3.7 MBq) was injected IP and radiotracer uptake was measured using PET/CT imaging (see below for specifics). Tumor burden was measured through detection of intravenously (IV) injected luciferin (GoldBio, 50 µL of a 20 mg/ml solution) and detected using an IVIS Spectrum In Vivo Imaging System (PerkinElmer). Total bioluminescent signal (BLI) was normalized to the measurement taken on day 2 and displayed as fold change in BLI. BLI measurements were taken every five days until mice reached endpoint.

To evaluate the sensitivity of the huC825-19BBz *in vivo*, CAR T-cells were stimulated with OKT3 *in vitro* overnight prior to implant. 1x10^4, 3x10^4, 1x10^5 and 3x10^5 huC825-19BBz cells were implanted subcutaneously over the left and right shoulder in 40 µL of 50:50 RPMI:Matrigel solution. [^{86}Y]Y-ABD (3.7 MBq) was injected via tail vein and the radiotracer uptake was measured 3 hours post tracer injection by PET/CT.

A modified version of the Raji SC model was utilized to characterize the huC825-19BBz CAR-T cells on a tissue level. This model followed the same procedure as above with one exception: T cells were injected fourteen days after tumor engraftment. [^{86}Y]Y-ABD was injected on day 7 and 14 post T cell injection and measured as above.

Ex-vivo Flow Cytometry Time Course

NSG mice injected with huC825-19BBz CAR-T cells were sacrificed on d 4, d 7, d 11, and d 14 post T cell injection using the previously described Raji SC model. Tumors were collected and analyzed by flow cytometry to determine the ratio of T cells to tumor cells overtime. huC825-19BBz CAR-T cells were identified as $CD3^+$, $CD45^+$, CAR^+ using the same antibodies as detailed in the main text. Raji tumor cells were identified as GFP^+ and $CD20^+$ through use of an anti-CD20 Alexa647 antibody (BioLegend, 302318).

Image-Based Biodistribution

Volumes of interest (VOIs) were defined using a combination of manual and semi-automatic segmentation techniques (VivoQuant or 3D Slicer v4.10); CT anatomical guidance was used to segment the blood/heart contents, lungs, liver, stomach contents, intestine contents, kidneys, skeleton, muscle, and tumor tissue. PET guidance was used to determine activity within the urinary bladder.

Dosimetry

Image co-registration, segmentation, and Monte Carlo dosimetry simulation. Dosimetry estimates were based on co-registered [^{86}Y]Y-ABD serial PET/CT images obtained at 14 d post T cell administration. The [^{86}Y]Y-ABD PET images were originally quantified in units of %IA/mL, decay corrected to time of administration (as-described previously) and therefore represent biological clearance over time. The CT scan acquired at the 16 h time point was used as the “fixed” volume to which the PET/CT images at other time points (1, 3, or 36 h) were co-registered. Tumor, organs-of-interest, and residual tissues were contoured using a combination of manual and semi-automatic segmentation techniques in 3D Slicer image analysis software.

After manual alignment of the CT images, rigid registration (3 rotational and 3 translational degrees of freedom) was performed, followed by B-spline deformable registration using the Plastimatch extension of 3D Slicer. The deformable registration step used a mean squared error

cost function, a 2x2x2 image subsampling rate, a 7.5 mm grid size, and was performed in a single stage. After successful co-registration of the CT images with the 16 h CT, the transforms were applied to the respective PET images. The PET images were then resampled to the same image dimensions, spacing, and origin. The resampled [^{86}Y]Y-ABD PET images were then used forecast the effective activity concentration (i.e., representing biological clearance and physical decay) of therapeutic analogs – [^{177}Lu]Lu-ABD, [^{90}Y]Y-ABD, or [^{225}Ac]Ac-Pr – under the assumption that biological clearance kinetics are equivalent among these variants, viz:

$$\left[\left(\frac{\%\mathrm{IA_e}}{\mathrm{mL}}\right)_{\mathrm{I}}\right]_{\mathrm{i,j,k}}(\mathrm{t}) = \left[\left(\frac{\%\mathrm{IA_b}}{\mathrm{mL}}\right)_{\mathrm{Y\text{-}86}}\right]_{\mathrm{i,j,k}}(\mathrm{t}) \times \mathrm{e}^{-\lambda_{\mathrm{I}}\mathrm{t}}$$

where $\left[\left(\frac{\%\mathrm{IA_b}}{\mathrm{mL}}\right)_{\mathrm{Y\text{-}86}}\right]_{\mathrm{i,j,k}}(\mathrm{t})$ is the decay-corrected activity concentration of [^{86}Y]Y-ABD in the voxel indexed by i, j, and k; λ_{I} is the physical decay constant for radionuclide I (i.e., [^{177}Lu]Lu-ABD, [^{90}Y]Y-ABD, or [^{225}Ac]Ac-Pr); t is the time post-administration; and, $\left[\left(\frac{\%\mathrm{IA_e}}{\mathrm{mL}}\right)_{\mathrm{I}}\right]_{\mathrm{i,j,k}}(\mathrm{t})$ is the forecasted activity concentration of radionuclide I. A time-integrated activity coefficient image (in units of Bq*s/Bq*mL) was obtained for each radionuclide as follows: the $\left[\left(\frac{\%\mathrm{IA_e}}{\mathrm{mL}}\right)_{\mathrm{I}}\right]_{\mathrm{i,j,k}}(\mathrm{t})$ was voxel-wise integrated in time via the trapezoidal method up to the last measured time point, after which clearance was assumed to occur by physical decay only. The $\left[\left(\frac{\%\mathrm{IA_e}}{\mathrm{mL}}\right)_{\mathrm{I}}\right]_{\mathrm{i,j,k}}(\mathrm{t})$ at $\mathrm{t} = 0$ was assumed to be equal to the $\left[\left(\frac{\%\mathrm{IA_e}}{\mathrm{mL}}\right)_{\mathrm{I}}\right]_{\mathrm{i,j,k}}(\mathrm{t})$ at the first measured time point. The time-integrated images were used as the voxel source term in the PHITS Monte Carlo simulations used to compute the equivalent dose coefficients for [^{177}Lu]Lu-ABD or [^{90}Y]Y-ABD. The simulation geometry was defined by the segmentation described above. Where available, each segment was assigned material composition and density defined by the International Commission on Radiological Protection (ICRP) Publication 89; in case a specific tissue was unavailable in the ICRP tables, the elemental composition and density of soft tissue was used. For [^{225}Ac]Ac-Pr, a

Monte Carlo simulation was not run; the equivalent dose coefficients [Sv/mCi administered] were computed by assuming local energy deposition from radiations produced by ^{225}Ac and all radioactive progenies. In all cases, a radiation weighting factor of unity for beta particles/photons was used for weighting the absorbed doses to obtain equivalent dose coefficients [Sv/mCi administered]. For alpha particles, a radiation weighting factor of 5 was used (which reflects the relative biological effectiveness of alpha particles for deterministic endpoints).

Estimated dosimetry for human administration. Murine organ-level time integrated activity coefficients [Bq*s/Bq] were derived as the product of the corresponding segment volumes and the mean time-integrated activity concentration, normalized to the administered activity (vide supra). The organ-level level time-integrated activity coefficients were assumed, in first order, to be independent of species, and were input into MIRDcalc dosimetry software [3] to obtain absorbed dose estimates for humans using the ICRP adult reference phantoms.

Autoradiography and Immunohistochemical Staining

Tissues were frozen in OCT (Sakura Finetek USA, Inc.) immediately after collection, sectioned at 5 microns thickness, mounted on slides and fixed in 10% neutral buffered formalin (NBF) prior to staining. Other tissues were fixed in 10% NBF, processed in alcohol and xylene, embedded in paraffin, sectioned at 5 microns thickness and mounted on slides prior to staining. Immunohistochemistry was performed on a Leica Bond RX automated stainer using Bond reagents (Leica Biosystems, Buffalo Grove, IL), including a polymer detection system (DS9800, Novocastra Bond Polymer Refine Detection, Leica Biosystems, Buffalo Grove, IL). The chromogen was 3,3 diaminobenzidine tetrachloride (DAB), and sections were counterstained with hematoxylin. After heat-induced epitope retrieval in a citrate buffer (performed for formalin-fixed paraffin embedded sections only), staining was performed with a primary antibody against CD3 (clone SP162, Abcam ab135372) applied at a concentration of 1:200. Adjacent sections were

hematoxylin-and eosin-stained for morphologic evaluation. Stained slides were evaluated by a board-certified veterinary pathologist (Laboratory of Comparative Pathology, MSKCC).

For the enumeration of T cells in selected tissue and tumor sections, $CD3^+$ cell density maps were created with a diameter of 0.5 µm per cell nucleus using the software Qupath v0.3.2. To get an estimation of the total number in a tissue, the average of 10 sections was extrapolated to the full tumor volume.

TABLE S1. Specific and non-specific binding of huC825 expressing T cells with [^{111}In]In-Pr. B_{max}, sites/cell; K_d, equilibrium dissociation constant, [nM]. Representative data shown. Experiments were performed in triplicate at 37 °C. n = 3 donors.

One site – Total and non-specific binding			
	**huC825s-19BBz**	**huC825-19BBz**	**huC825-GFP**
B_{max}	25000 ± 17000	76000 ± 48000	37000 ± 14000
K_d	0.72 ± 0.40	0.72 ± 0.35	0.16 ± 0.02
R^2	0.98 ± 0.01	0.99 ± 0.01	0.98 ± 0.01

TABLE S2. Quantification of $CD3^+$ T-cells in selected tissues. Tissues of selected organs and tumor tissue from Figure 3A. Absolute number of $CD3^+$ cells were counted on QuPath v 0.3.0 and absolute number of $CD3^+$ cells per mm^3 were calculated, leading to an estimation of the total amount of $CD3^+$ cells.

Tissue	Tissue Volume [mm^3]	$CD3^+$ cells/mm^3	Total $CD3^+$ cells
**Lungs**	578.58	6,642.00	3,842,974
**Primary tumor**	3,991.46	2,731.70	10,903,471
**Liver metastasis**	1.67	12,671.00	21,160
**Liver (excluding metastasis)**	1,705.07	2,720.40	4,638,472
**Spleen**	79.20	20,601.00	1,631,642
**Muscle**	759.95	4.39	3,338

TABLE S3. Projected murine [^{86}Y]Y-ABD doses. Absorbed dose per organ or tissue was calculated using the dosimetry described in Figure 5.

Tissue/ Area of interest	Absorbed dose [Gy/mCi]	Absorbed dose [Gy/MBq]
Tumor	4.92	0.133
Heart	3.82	0.103
Lungs	5.96	0.161
Liver	4.88	0.132
Spleen	4.22	0.114
Kidneys	3.61	0.098
Skeleton	1.34	0.036
Rest of body	1.95	0.053

TABLE S4. Dose estimates for huC825-19BBz and 19BBz T cells

huC825-19BBz<br>Summary RBE-weighted doses (Sv/mCi)				
**Segment**	**177Lu**	**90Y**	**86Y**	**225Ac**
Cortical bone	2.89	9.78	1.34	4397
Lungs	37.3	57.9	5.96	65138
Heart	14.8	40.4	3.82	22890
Tumor	21.2	52.0	4.92	32526
Rest of body	4.21	13.1	1.95	6264
Kidneys	15.8	36.0	3.61	24310
Spleen	18.9	40.4	4.22	29091
Liver	18.6	49.3	4.88	28187

CAR<br>Summary RBE-weighted doses (Sv/mCi)				
**Segment**	**177Lu**	**90Y**	**86Y**	**225Ac**
Cortical bone	0.0458	0.349	0.125	47.2
Lungs	0.204	0.858	0.255	242
Heart	0.129	0.710	0.209	161
Tumor	0.0624	0.425	0.142	63.3
Rest of body	0.160	0.870	0.251	185
Kidneys	1.58	3.66	0.521	2392
Spleen	0.328	1.85	0.443	385
Liver	0.663	2.26	0.456	937

Table S5. Pathological assessment of [^{225}Ac]Ac-Pr long-term toxicity study. Non-tumor-bearing NSG mice were injected with 2 μCi and 4 μCi [^{225}Ac]Ac-Pr (n = 3) and one representative mouse was assessed by a pathologist 150 days post tracer injection, N: normal; MF: multifocal; D: Diffuse; 1: minimal; 2: mild; 3: moderate; 4: marked; U: unavailable

**Signalment**		
**Sex**	**Female**	**Female**
**Age**	**30 weeks**	**30 weeks**
**Species**	**Mouse**	**Mouse**
**Strain**	**NSG**	**NSG**
**Experimental history**		
**Treatment**	IV administration of 2 μCi of [^{225}Ac]Ac-Pr. Necropsy 150 days post-administration.	IV administration of 4 μCi of [^{225}Ac]Ac-Pr. Necropsy 150 days post-administration.
**Histologic examination**		
**Heart**	Tissues show mild to moderate post-mortem autolysis.	Tissues show mild to moderate post-mortem autolysis.
**Heart**	N	N
**Lungs**	N	N
**Thymus**	N	N
**Kidneys**	N	N
**Liver**	Hepatocyte apoptosis, random, MF, 1. Hepatocyte mitoses, random, MF, 1. Hepatocyte karyocytomegaly, D, 1; Extramedullary hematopoiesis, 1.	Hepatocyte apoptosis, random, MF, 1. Hepatocyte mitoses, random, MF, 1. Hepatocyte karyocytomegaly, D, 2; Extramedullary hematopoiesis, 1.
**Gallbladder**	N	N
**Stomach**	N	N
**Duodenum, jejunum, ileum**	N	N
**Cecum**	N	N
**Colon**	N	N
**Mesenteric lymph node**	U	N
**Salivary glands**	N	N
**Uterus**	N	N
**Cervix**	N	N
**Vagina**	N	N
**Urinary bladder**	N	N
**Spleen**	N	N
**Pancreas**	N	N
**Adrenals**	N	N
**Ovaries**	N	N
**Oviducts**	N	N
**Trachea**	N	N
**Esophagus**	N	N
**Thyroid**	N	N
**Skin (trunk, head)**	N	Acanthosis and hyperkeratosis, with intracorneal bacteria, MF, 4.

**Mammary glands**	N	N
**Bones (femur, tibia, sternum, vertebrae)**	N	N
**Bone marrow (femur, tibia, sternum, vertebrae)**	N	N
**Stifle joint**	N	N
**Skeletal muscles (hind limb, spine)**	N	N
**Nerves (hind limb, spine)**	N	N
**Spinal cord**	N	N
**Oral cavity**	N	N
**Teeth**	N	N
**Nasal cavity**	N	N
**Eyes**	N	N
**Harderian gland**	N	N
**Bones (skull)**	N	N
**Pituitary**	N	N
**Brain**	N	N
**Ears**	N	N

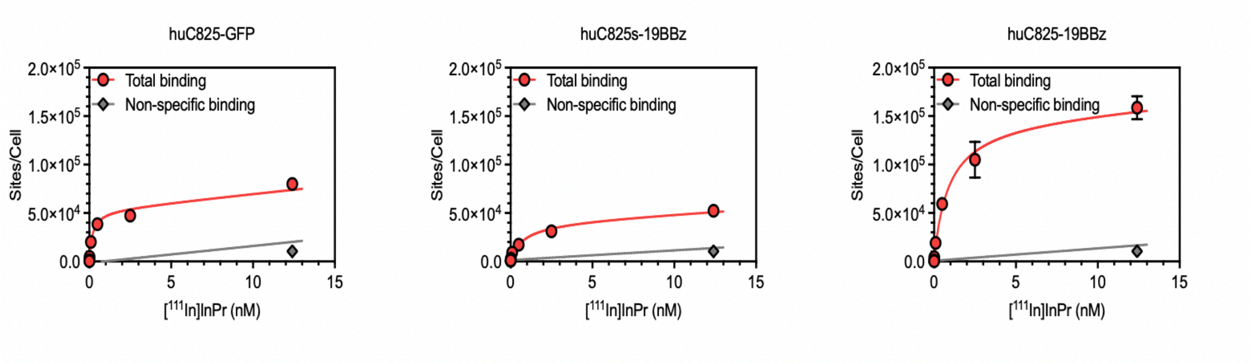

FIGURE S1. Saturation binding curve of [^{111}In]In-Pr to huC825-GFP, huC825s-19BBz and huC825-19BBz T-cells. Representative data shown. Experiments were performed in triplicate at 37 °C. n = 3 donors.

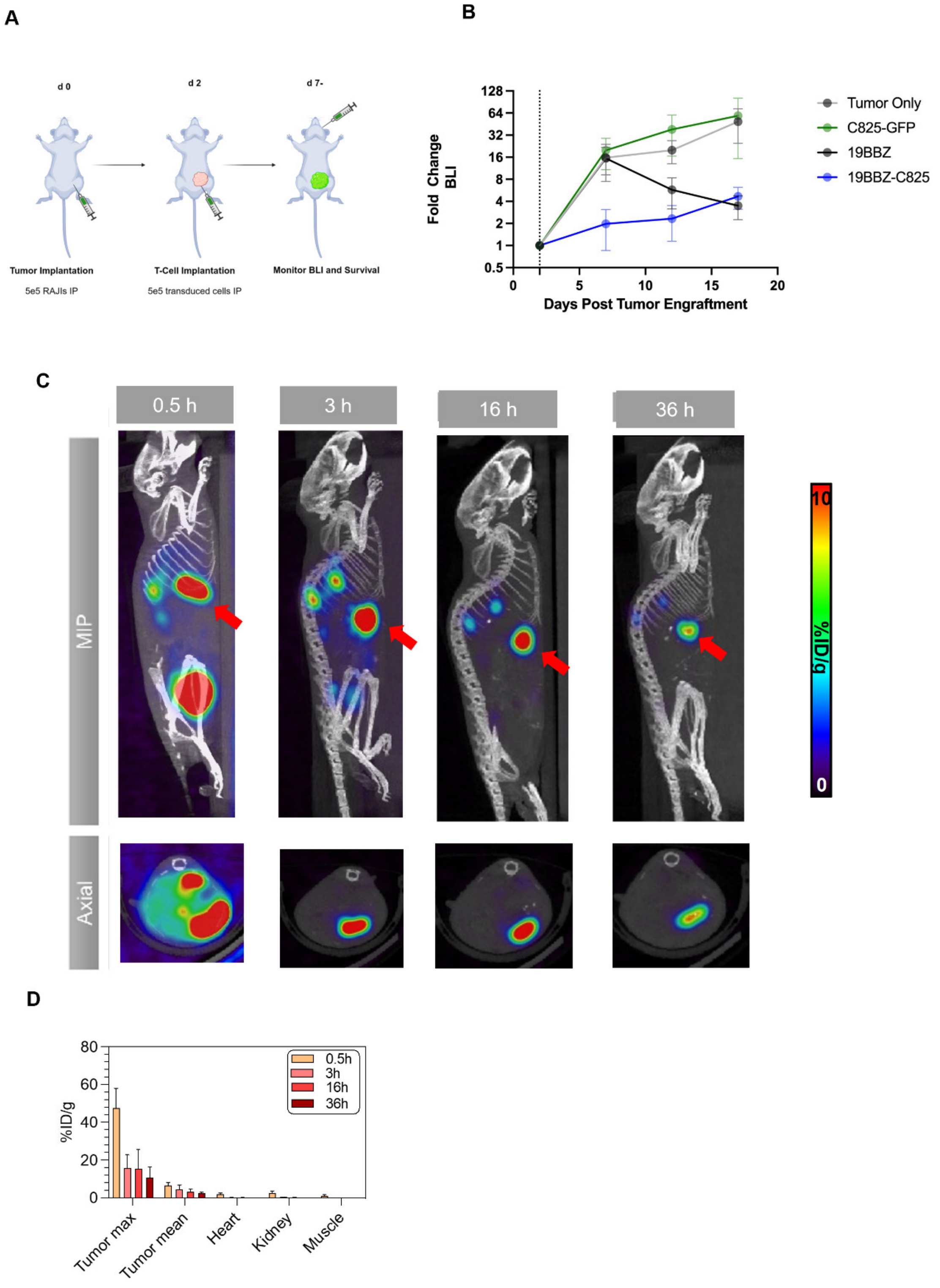

FIGURE S2. Thor-cells can be visualized intraperitoneally and maintain CAR-mediated cytotoxic capability *in vivo*. (A) Raji cells ($5x10^5$) were implanted intraperitoneal (IP) into NSG mice, injected with CAR-T cells ($5x10^5$) IP at day 2. (B) Tumor growth is displayed as fold change of bioluminescence signal from day of CAR T injection (mean ± SE is depicted; (d 17: p = 0.07 for Tumor only vs 19BBZ, p = 0.07 for Tumor only vs 19BBz-C825, unpaired, two-tailed t-test, n = 6).

(C) Maximum intensity projection PET/CT images at d 14 post huC825-19BBz administration, imaged with PET/CT at 0.5, 3, 16, and 36 h post-injection of radiotracer [^{86}Y]Y-ABD demonstrate specific uptake at the tumor site (red arrow), n = 4 (D) Image-based biodistribution. For tumor $[\%ID/g]_{max}$ and $[\%ID/g]_{mean}$ and for normal tissues $[\%ID/g]_{mean}$ are provided. (mean ± SD, n = 4)

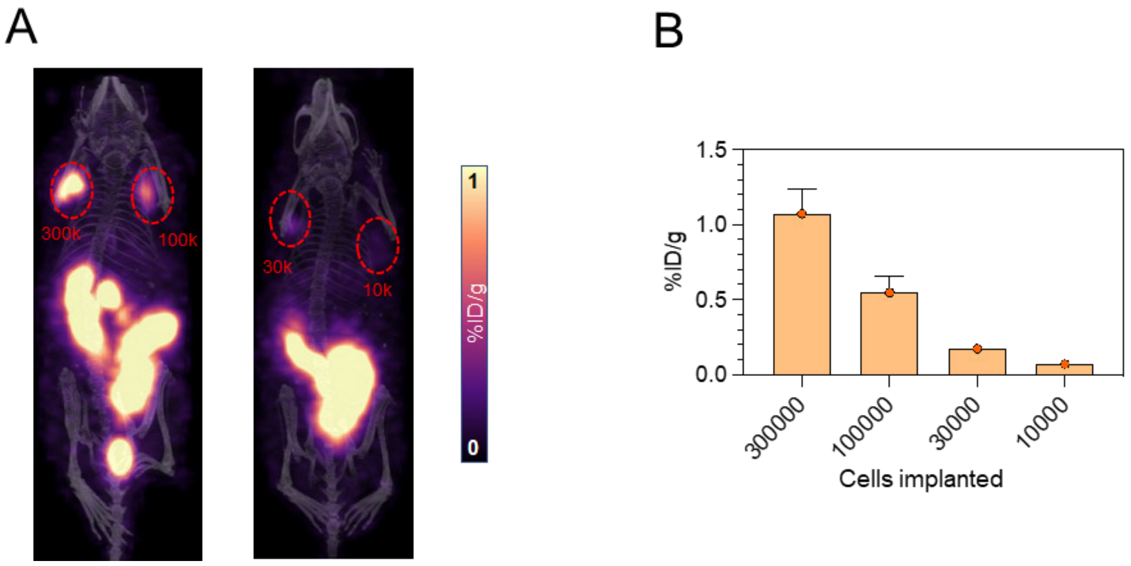

FIGURE S3. Sensitivity of huC825-19BBz CAR-T cells *in vivo*. (A) 19BBz-huC825 T cells ($3x10^5$,$1x10^5$, $3x10^4$,$1x10^4$) were implanted subcutaneously into NSG mice and imaged by PET/CT 3 h post intravenous radiotracer ([^{86}Y]Y-ABD) injection. Maximum-intensity projection (MIP) and axial images of representative mouse shown (n = 4) (B) Image-based biodistribution. [%ID/g]mean of CAR T cell implant is provided. (mean ± SD, n = 4)

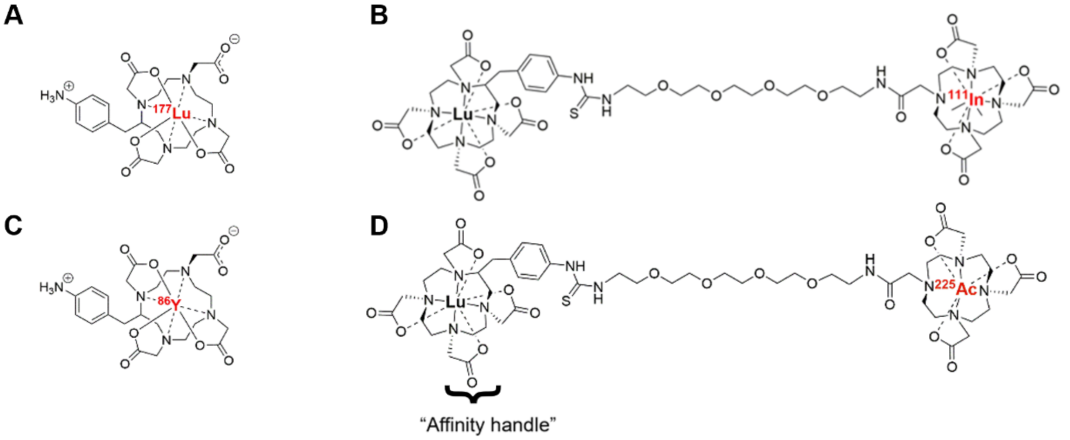

FIGURE S4. Chemical structures of radiohaptens. (A) [^{177}Lu]Lu-ABD. (B) [^{111}In]In-Pr. (C) [^{86}Y]Y-ABD. (D) [^{225}Ac]Ac-Pr.

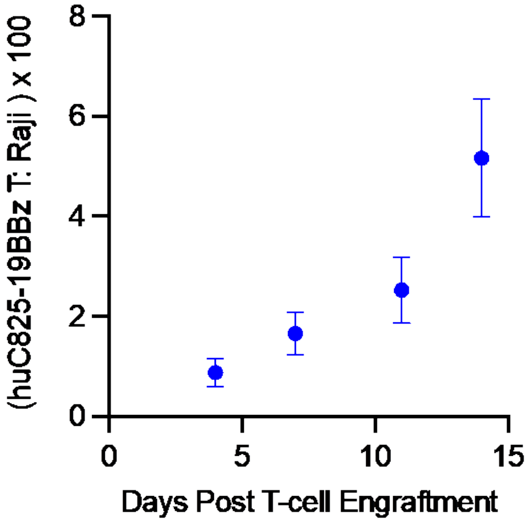

FIGURE S5. *Ex-vivo* flow cytometry quantification of intra tumoral huC825-19BBz CAR-T cells. Raji cells (3x10^6) were implanted subcutaneously into NSG mice, injected with huC825-CAR (3x10^6) intravenously seven days later, and tumors were collected and analyzed at d 4, d 7, d 11 and d 14 post T cell injection. The ratio of huC825-19BBz cells (identified as $CD3^+$,$CD45^+$, and CAR^+) to Raji tumor cells is displayed.

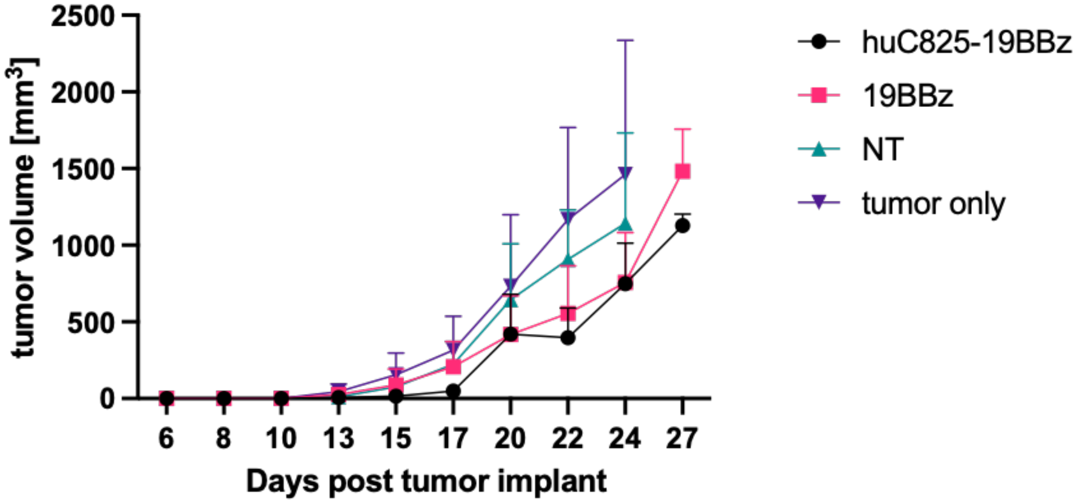

FIGURE S6. Subtherapeutic CAR-T cell model – no difference between 19BBz and Thor-cells. Raji cells (3x10^6) were implanted subcutaneously into NSG mice, injected with huC825-19BBz, 19BBz or non-transduced (NT) T cells (3x10^6) intravenously seven days later. Tumor growth is displayed as tumor volume measured by caliper (mean + s.e. is depicted; n = 5)

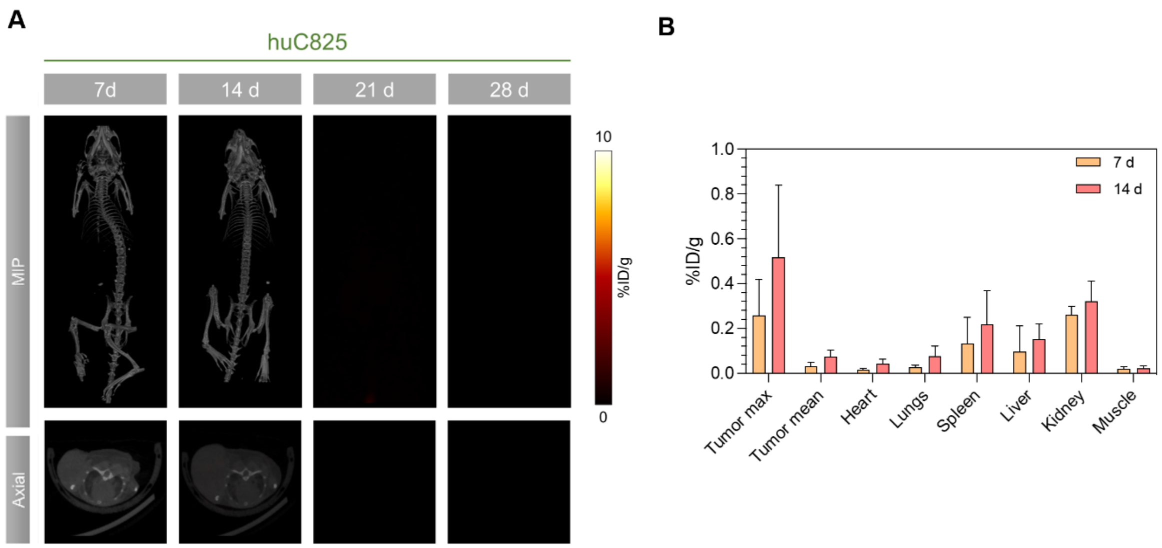

FIGURE S7. Radiotracer uptake in huC825-GFP group. (A) Raji cells (3x10^{6}) were implanted subcutaneously into NSG mice, injected with huC825-GFP T cells (3x10^{6}) intravenously seven days later and imaged by PET/CT on d 7, d 14 at 16 h post intravenous radiotracer ([^{86}Y]Y-ABD) injection (mice reached endpoint at d 18). Maximum intensity projection (MIP) and axial images of representative mouse shown at d 7 and d 14. (n = 4) (B) Image-based biodistribution. For tumor [%ID/g]$_{max}$ and [%ID/g]$_{mean}$ and for normal tissues [%ID/g]$_{mean}$ are provided. (mean ± SD, n = 4)

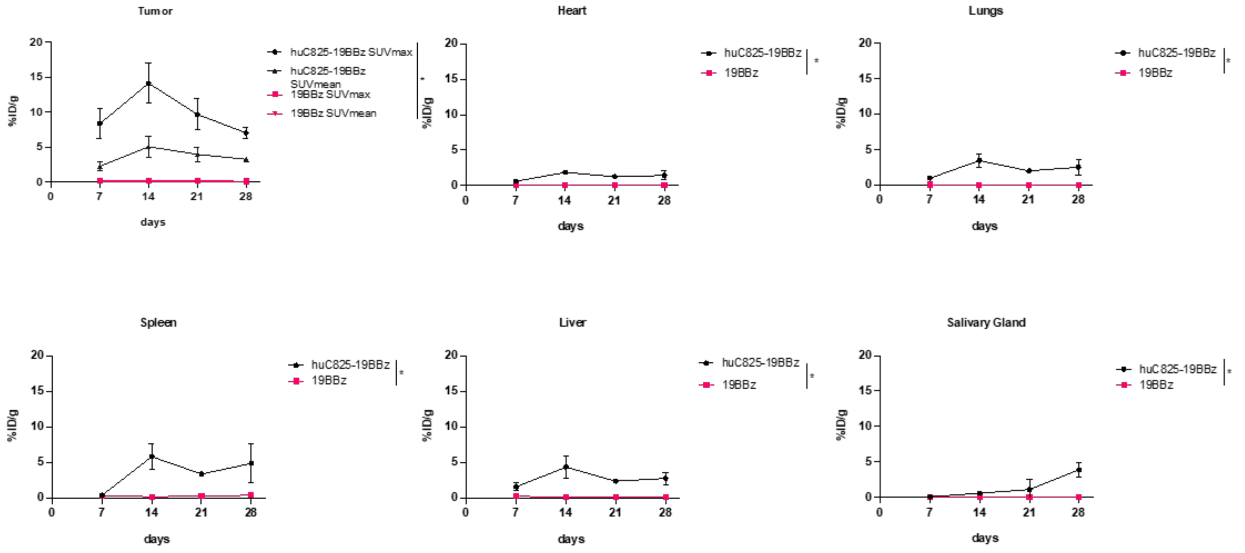

FIGURE S8. Time-activity curves of [^{86}Y]Y-ABD serial imaging study. Image-based biodistribution of [^{86}Y]Y-ABD in Raji-bearing NSG mice after administration of huC825-19BBz or 19BBz at d 7, d 14, d 21 and d 28 imaged at 16 h post [^{86}Y]Y-ABD administration i.v. (mean ± SD, n = 3, *: $p < 0.05$)

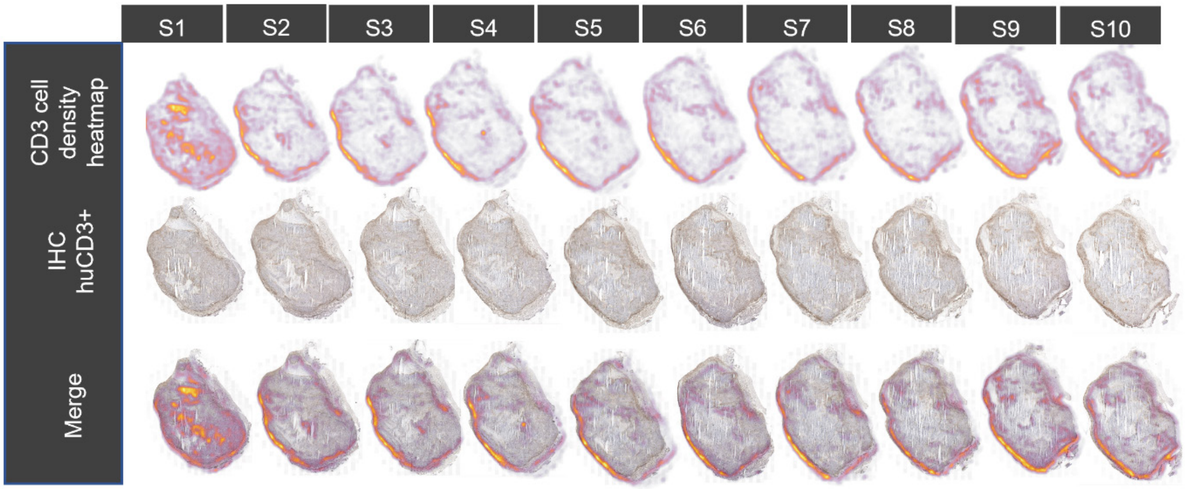

FIGURE S9. THOR cell quantification based on CD3⁺ immunohistochemistry. CD3 immunohistochemistry performed on tumor tissue harvested on day 28 post T cell injection confirmed infiltration of Thor cells. $CD3^+$ cell density maps generated with QuPath v.0.3.2; T cell number calculated as the average of 10 representative slices of tumor tissue.

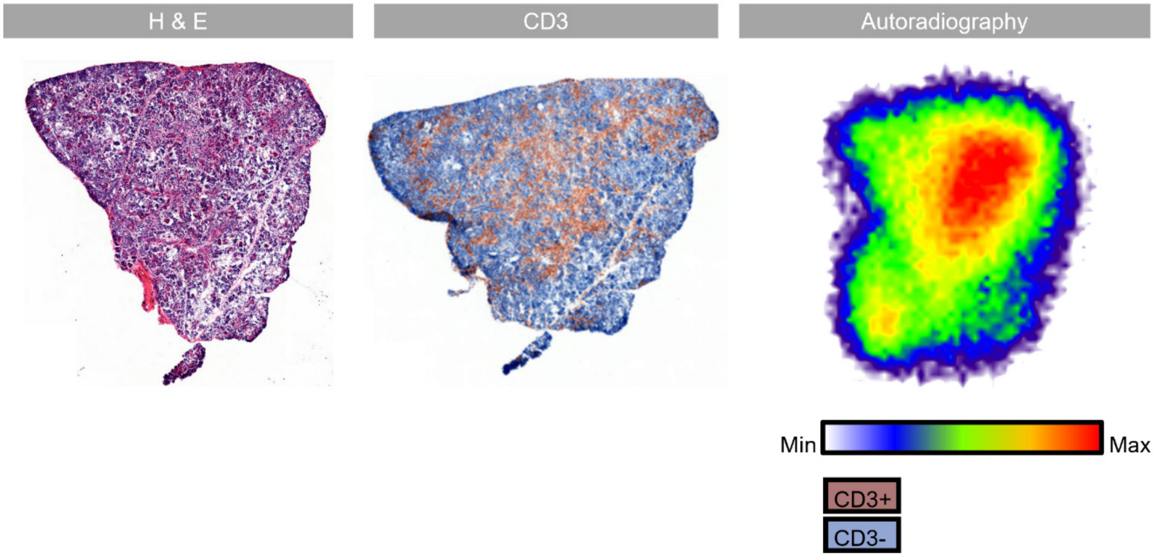

FIGURE S10. Immunohistochemistry and autoradiography of salivary gland. Autoradiography and immunohistochemistry of salivary gland tissue containing huC825-19BBz T cells confirm localization to off-target tissue. Radiotracer co-localizes with CD3-positive T cell cluster (brown staining). H&E = hematoxylin and eosin.

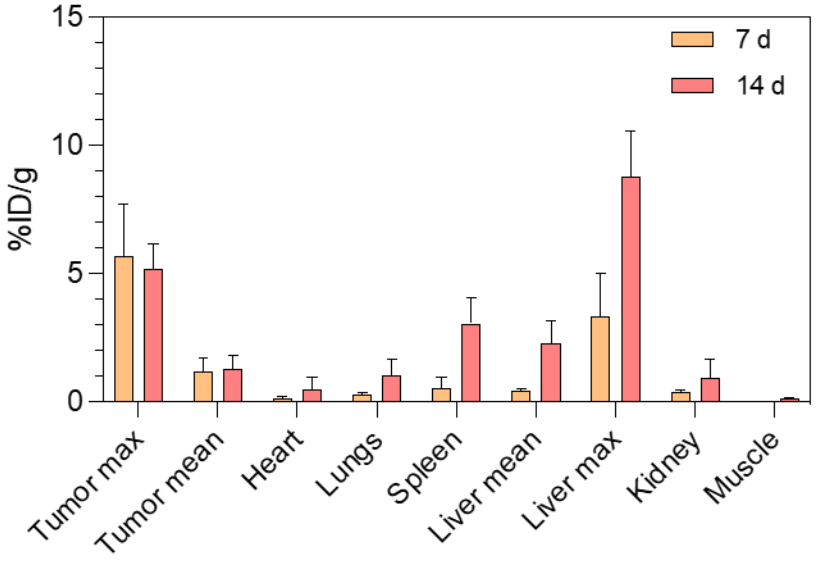

FIGURE S11. Image-based biodistribution. Image-based biodistribution for Figure 3B. For tumor and liver including secondary tumor sites $[\%ID/g]_{max}$ and $[\%ID/g]_{mean}$ and for normal tissues $[\%ID/g]_{mean}$ are provided. (mean ± SD, n = 4)

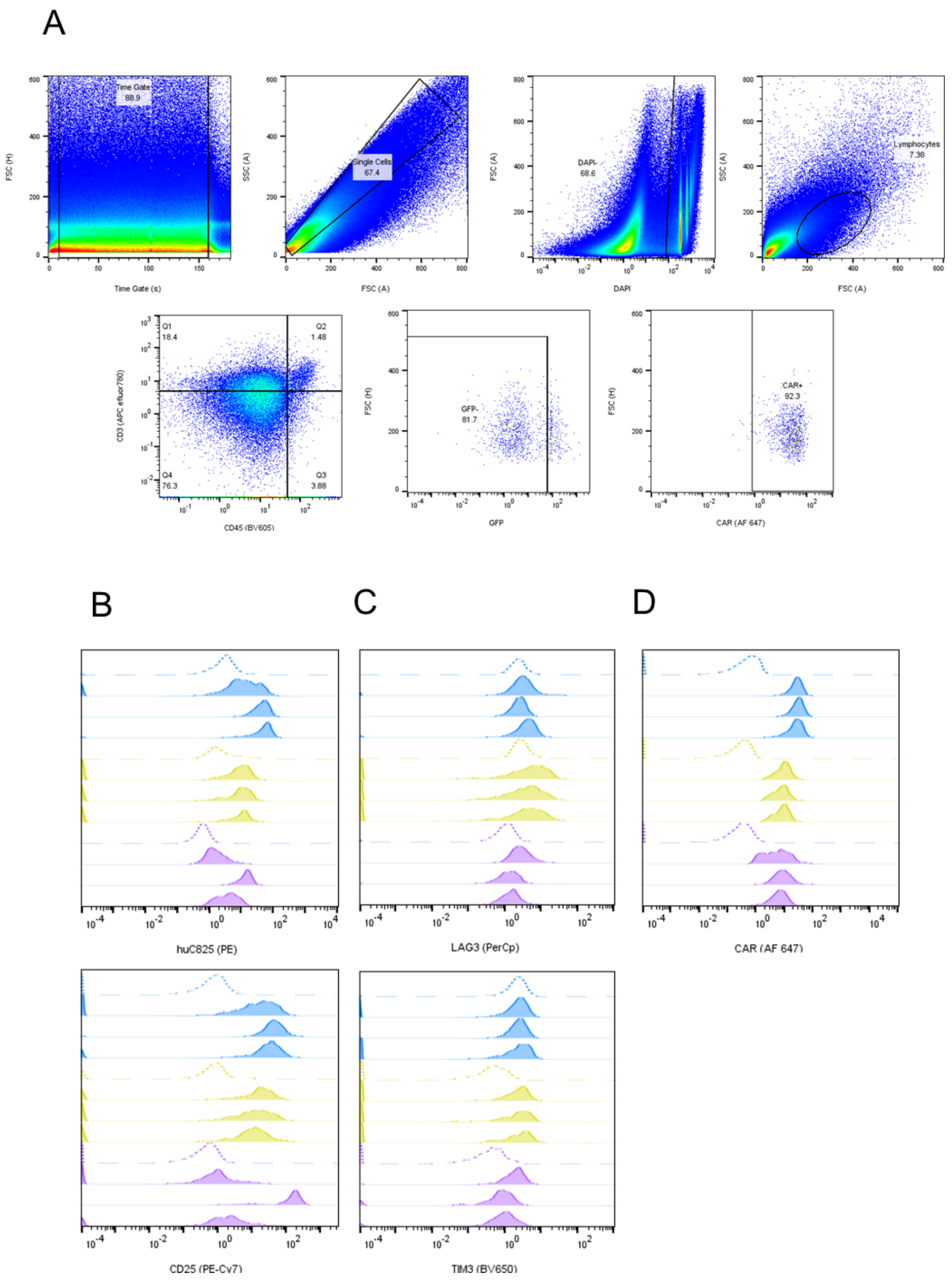

FIGURE S12. Raw Data for *ex-vivo* flow cytometry. (A) Gating strategy for data shown in Figure 4C-F. The gating strategy is as follows: time gate, doublet exclusion, viable cells (DAPI$^-$), lymphocytes, CD45$^+$ and CD3$^+$ cells, non-tumor cells (GFP$^-$), and CAR$^+$ cells. (B) Histogram of huC825 and CD25 data presented in Figure 4C & 4D. (C) Histogram of LAG3 and TIM3 data presented in Figure 4E & 4F. (D) Histogram of the CAR staining is presented. For (B-D): purple = spleen, yellow = tumor, blue = whole liver. Dashed unfilled histograms represent FMO's.

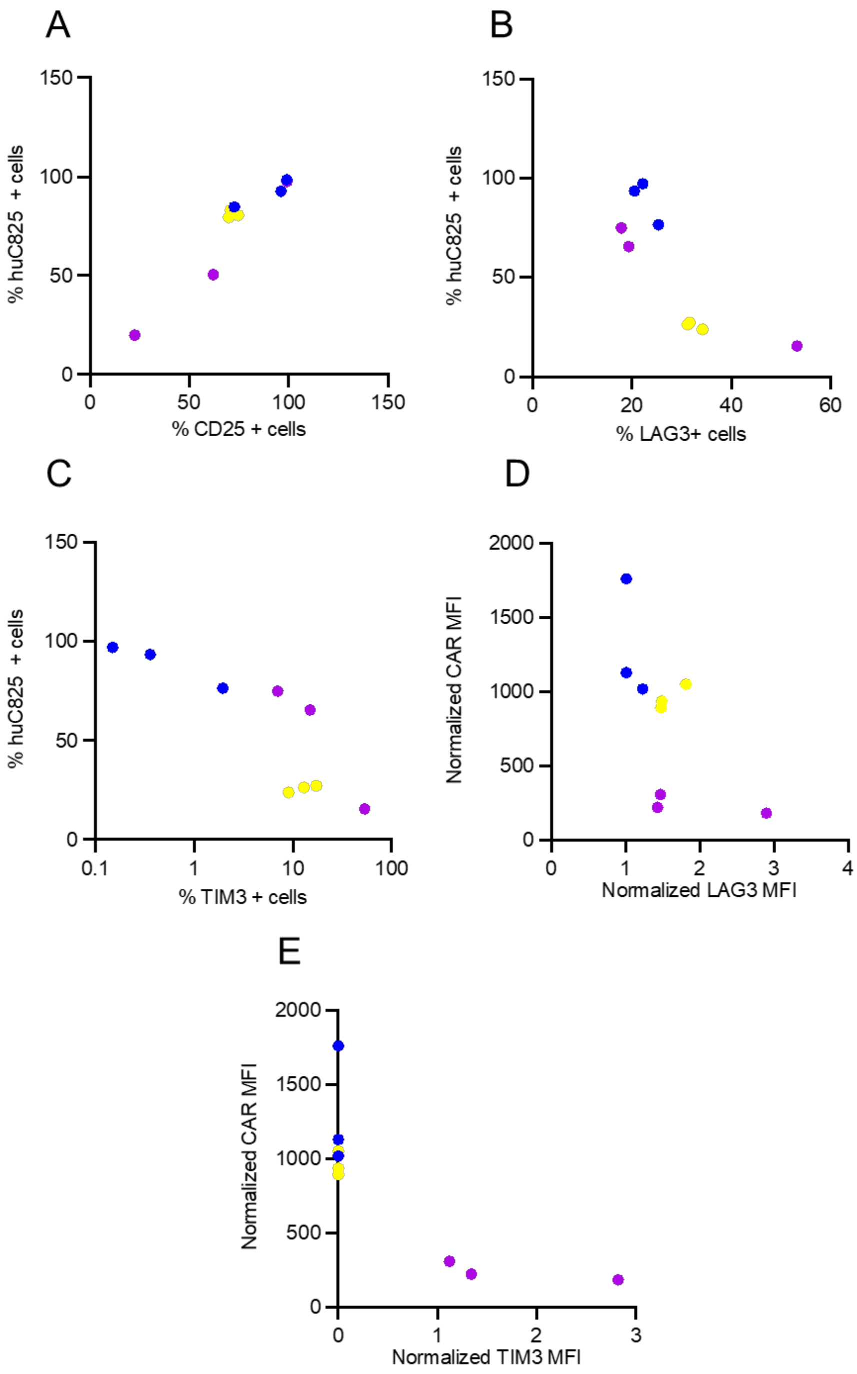

FIGURE S13. Correlation of percent positive huC825 cells with percent positive CD25, TIM3, and LAG3 cells and CAR MFI with exhaustion marker MFI. (A-C) Correlation analysis of gated huC825⁺ cells against gated CD25⁺ (A), LAG3⁺ (B), or TIM3⁺ (C) cells from the same samples as Figure 4C-F. (CD25: R^2=0.9333, p = 0.0007, LAG3: R^2=-0.7167, p = 0.039, TIM3: R^2=-0.85, p = 0.0061). (D-E) Correlation analysis of CAR MFI against LAG3 (D) or TIM3 (E) MFI from the same samples as Figure 4E-F. (LAG3: R^2=-0.5774, p = 0.1104, TIM3: R^2=-0.9143, p = 0.0016). For (A-E): purple=spleen, yellow=tumor, blue=whole liver.

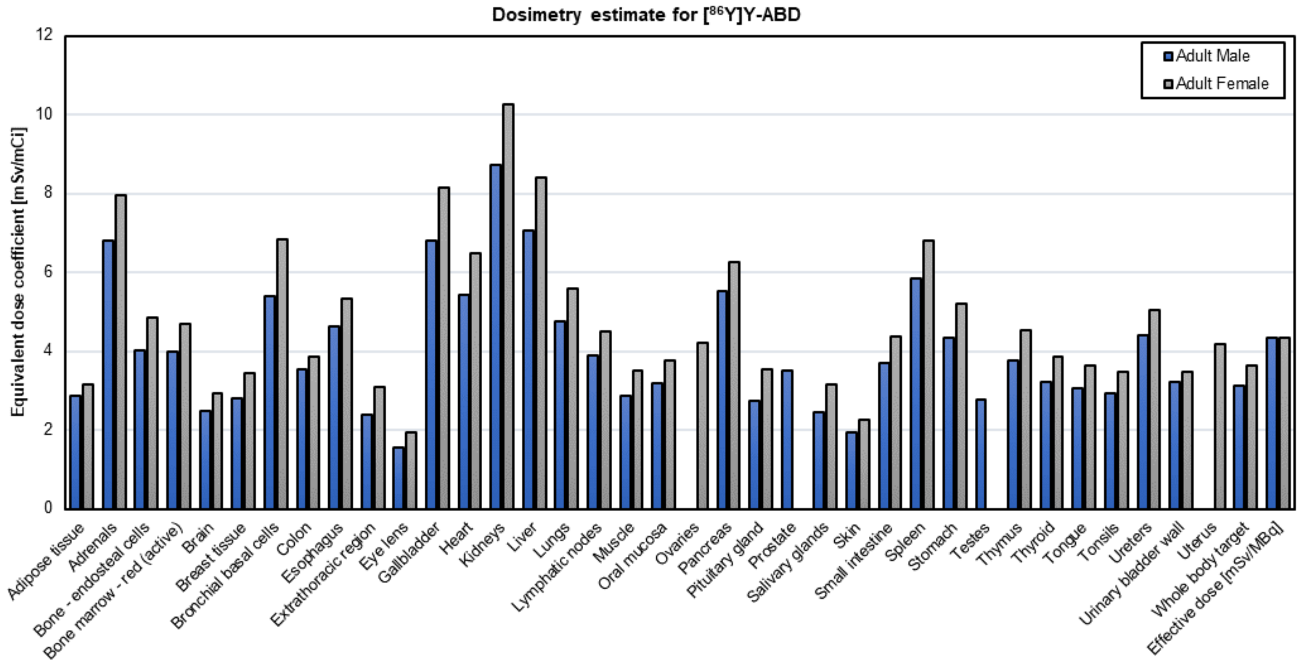

FIGURE S14. Human organ level dosimetry estimates extrapolated from murine biodistribution. Equivalent dose and effective dose coefficients estimated for reference adults for administration.

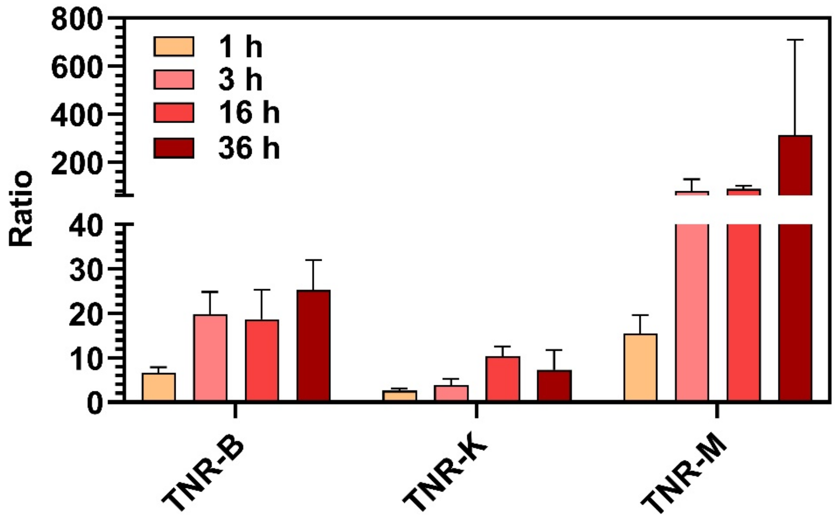

FIGURE S15. Favorable biodistribution with rapid clearance through the kidneys results in exceptionally high contrast in PET imaging in mice receiving Thor-cells. Tumor-to-normal-tissue ratios calculated from values extrapolated from Figure 5 (mean ± SD, n = 3). TNR = tumor-to-normal-tissue ratio, B = blood, K = kidney, M = muscle.

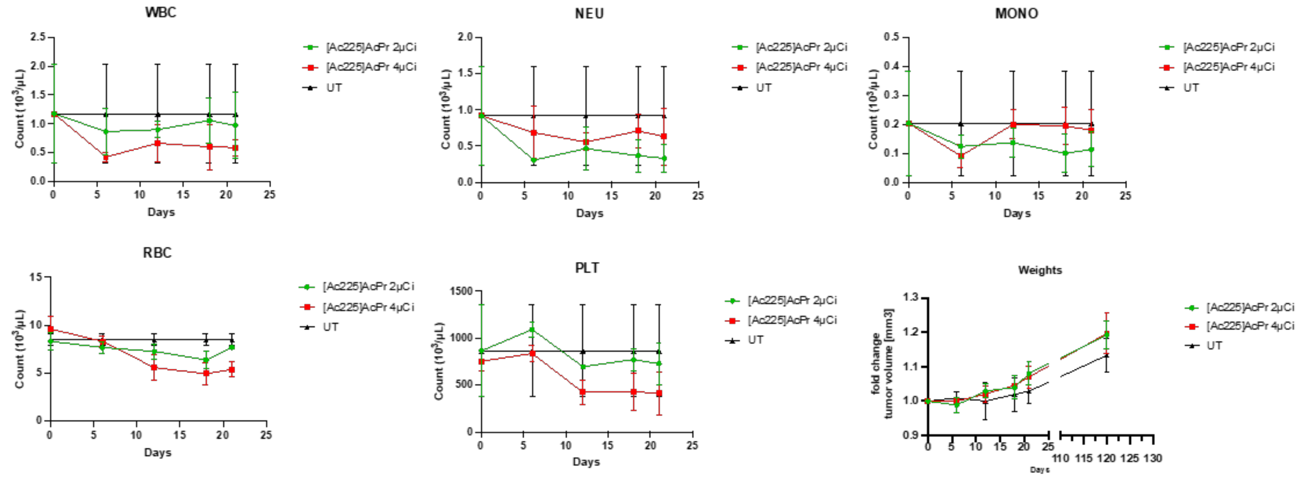

FIGURE S16. CBC analysis and weight measurements of non-tumor- bearing mice injected with [225Ac]Ac-Pr show no evidence of long-term toxicity. (A) CBC analysis from NSG mice intravenously injected with 2 µCi and 4 µCi of [225Ac]Ac-Pr with d 0 being the day of administration. Retroorbital blood collection was performed and measurement by HESKA hematocytometer. (B) Weight measurements indicate no long-term toxicity. $p > 0.1$. (mean ± SD, n = 3)

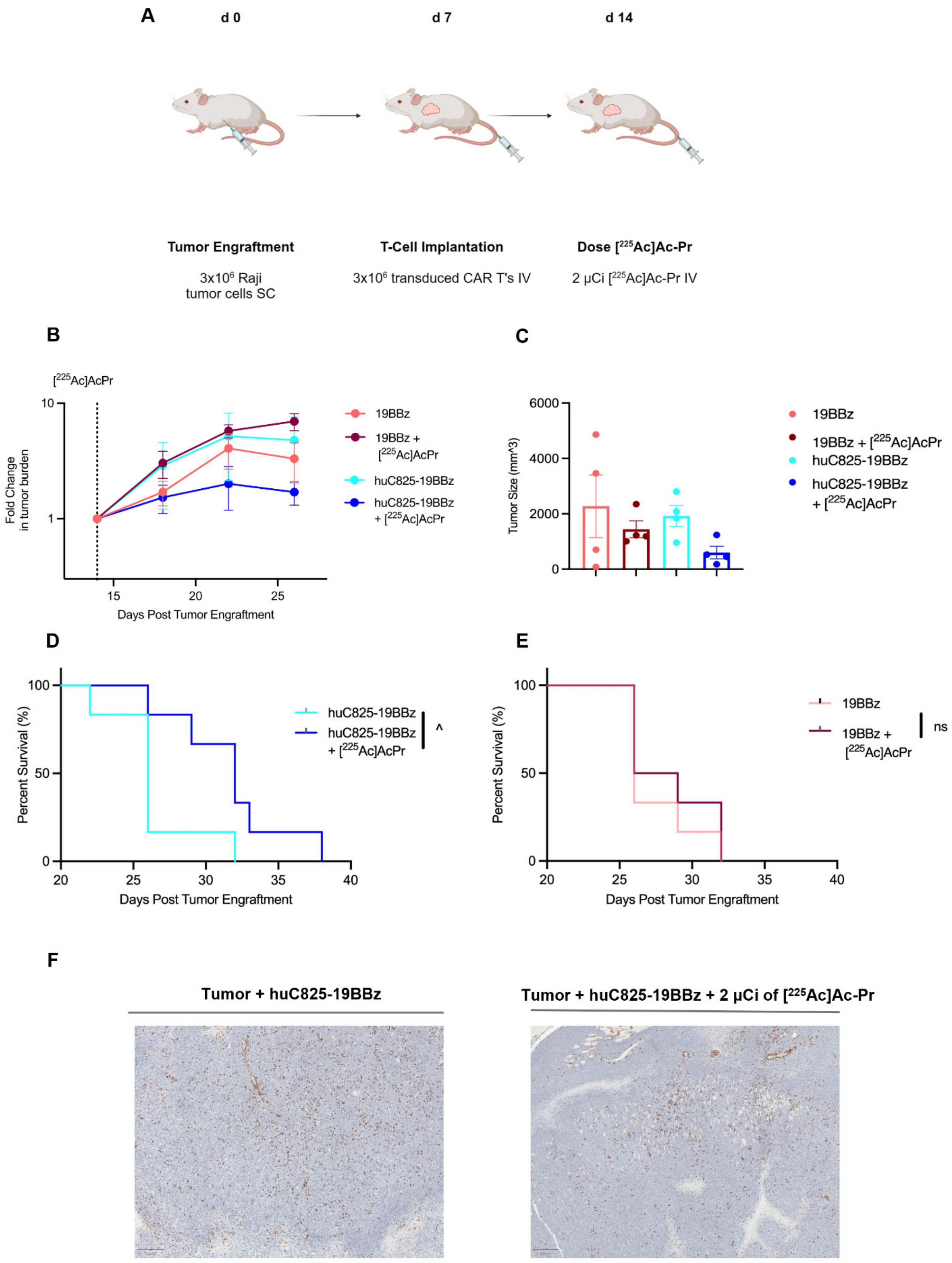

FIGURE S17. Thor-cells kill tumor cells in an orthogonal manner to their intrinsic T cell function. (A) Raji cells ($3x10^6$) were implanted subcutaneously into NSG mice, injected with

CAR-T cells (3x10^6) intravenously seven days post tumor engraftment, and injected intravenously with [^{225}Ac]Ac-Pr (74 kBq, 700 pmol) at d 7. (B) Tumor burden is displayed as fold change of bioluminescence signal from day of [^{225}Ac]Ac-Pr administration for one representative donor (n = 4). (C) Tumor volume measurements by caliper at d 26 post tumor engraftment (n = 4). (D, E) Kaplan-Meier survival curve of NSG mice injected with huC825-19BBz or 19BBz respectively after [^{225}Ac]Ac-Pr administration for two combined donors (n = 6). (F) $CD3^+$ IHC staining of tumor tissue untreated versus 14 d post [^{225}Ac]Ac-Pr injection. Data are displayed as ± SEM for (B) and (C). Two-tailed t-tests (*: $p < 0.05$) were utilized to test for differences between [^{225}Ac]Ac-Pr and untreated mice in (B) and (C). Log-rank Mantel-Cox was performed to test median differences in mouse survival (^: $p<0.05$).

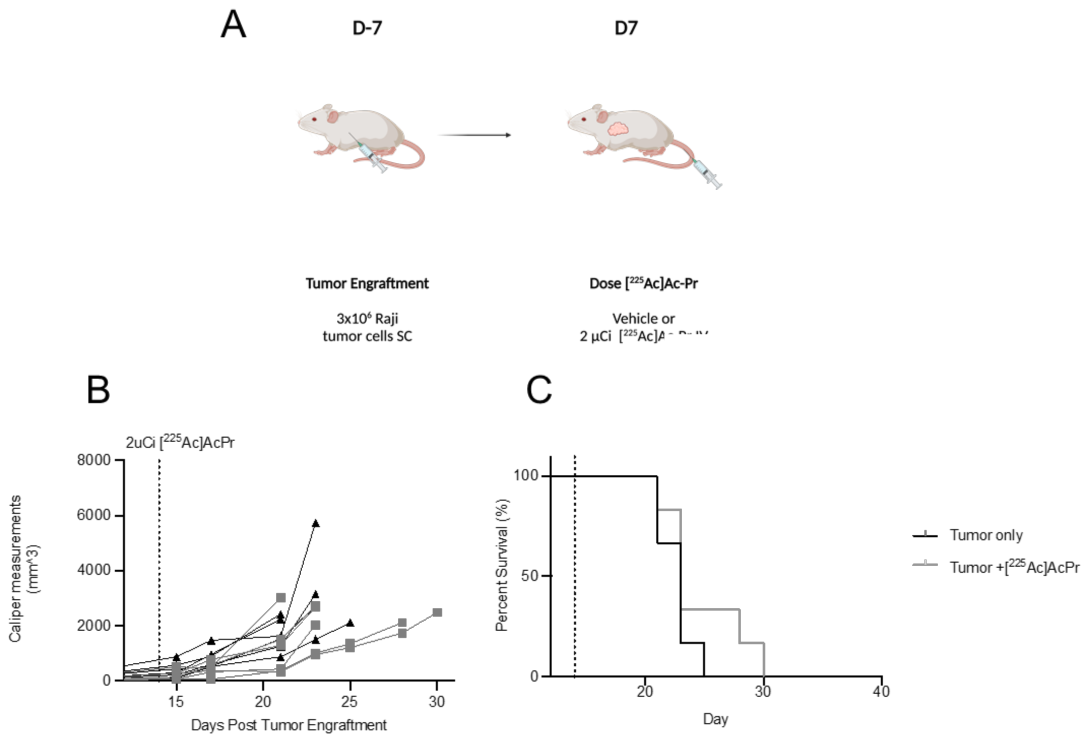

FIGURE S18. Tumor burden and survival curve analyses show [^{225}Ac]Ac-Pr has no significant anti-tumor effects as a monotherapy. (A) Raji cells (3x10^6) were implanted subcutaneously into NSG mice and injected intravenously with [^{225}Ac]Ac-Pr (2 µCi) 14 days after tumor implantation. (B) Tumor burden as measured by calipers from day of [^{225}Ac]Ac-Pr administration (n = 6). (C) Kaplan-Meier survival curve of NSG mice injected with [^{225}Ac]Ac-Pr compared to untreated mice (n = 6, p = 0.21).

References

1. Dacek MM, Veach DR, Cheal SM, et al. Engineered Cells as a Test Platform for Radiohaptens in Pretargeted Imaging and Radioimmunotherapy Applications. *Bioconjug Chem*. Apr 21 2021;32(4):649-654. doi:10.1021/acs.bioconjchem.0c00595

2. Aluicio-Sarduy E, Hernandez R, Valdovinos HF, et al. Simplified and automatable radiochemical separation strategy for the production of radiopharmaceutical quality (86)Y using single column extraction chromatography. *Appl Radiat Isot*. Dec 2018;142:28-31. doi:10.1016/j.apradiso.2018.09.016

3. Kesner AL, Carter LM, Ocampo JC, et al. MIRD Pamphlet No. 28, Part 1: MIRDcalc-A Software Tool for Medical Internal Radiation Dosimetry. *J Nuc Med* 2023 Jul;64(7):1117-1124. doi: 10.2967/jnumed.122.264225.
